# Role of main RNA modifications in cancer: N^6^-methyladenosine, 5-methylcytosine, and pseudouridine

**DOI:** 10.1038/s41392-022-01003-0

**Published:** 2022-04-28

**Authors:** Chen Xue, Qingfei Chu, Qiuxian Zheng, Shiman Jiang, Zhengyi Bao, Yuanshuai Su, Juan Lu, Lanjuan Li

**Affiliations:** State Key Laboratory for Diagnosis and Treatment of Infectious Diseases, National Clinical Research Center for Infectious Diseases, Collaborative Innovation Center for Diagnosis and Treatment of Infectious Diseases, The First Affiliated Hospital, College of Medicine, Zhejiang University, Hangzhou, 310003 China

**Keywords:** Gastrointestinal cancer, Gastroenterology

## Abstract

Cancer is one of the major diseases threatening human life and health worldwide. Epigenetic modification refers to heritable changes in the genetic material without any changes in the nucleic acid sequence and results in heritable phenotypic changes. Epigenetic modifications regulate many biological processes, such as growth, aging, and various diseases, including cancer. With the advancement of next-generation sequencing technology, the role of RNA modifications in cancer progression has become increasingly prominent and is a hot spot in scientific research. This review studied several common RNA modifications, such as N^6^-methyladenosine, 5-methylcytosine, and pseudouridine. The deposition and roles of these modifications in coding and noncoding RNAs are summarized in detail. Based on the RNA modification background, this review summarized the expression, function, and underlying molecular mechanism of these modifications and their regulators in cancer and further discussed the role of some existing small-molecule inhibitors. More in-depth studies on RNA modification and cancer are needed to broaden the understanding of epigenetics and cancer diagnosis, treatment, and prognosis.

## Introduction

Cancer is one of the main threats to human health worldwide.^1–3^ Over the past two decades, cancer incidence and mortality have been growing rapidly.^4,5^ In 2020, there was an estimated 19.3 million new cancer cases worldwide and nearly 10 million cancer deaths.^6^ Lung cancer has always been cancer with the highest worldwide incidence.^7–10^ However, the latest data suggest that new breast cancer (BRC) cases reached 2 million in 1 year.^11,12^ BRC incidence has surpassed lung cancer incidence and has become the first cause of global cancer.^13,14^ A series of reasons, such as environmental pollution,^15^ bad living habits,^16^ dietary structure,^17^ and population aging,^18^ lead to the emergence of this phenomenon. Although significant progress has been made in cancer treatment, such as surgery, radiotherapy, chemotherapy, immunotherapy, and biological therapy, the prognosis for many patients with cancer remains poor.^19,20^ Therefore, exploring the underlying molecular mechanisms of cancer occurrence and development is of great significance for the early identification of cancer and the establishment of new treatment options and is also crucial for improving the prognosis of cancer patients.

Epigenetic modification refers to a heritable change in the genetic material without any change in the nucleic acid sequence and results in a heritable phenotypic change.^21–23^ These changes included DNA methylation, histone modifications, chromatin remodeling, and RNA interference (RNAi).^24–26^ Epigenetic modification regulates many biological processes in the human body, such as growth,^27^ aging,^28^ and various diseases.^29–31^ DNA methylation, a form of DNA chemical modification, is the selective addition of methyl groups to DNA molecules under the action of DNA methyltransferase. DNA methylation can occur at the C-5 position of cytosine, the N-4 position of adenine, and the N-6 position of guanine.^32,33^ 5-Methylcytosine (m^5^C), the addition of a methyl group to cytosine, is the most common way of DNA modification in higher organisms in mammalian cells.^34–36^ m^5^C could effectively upregulate gene expression levels and inhibit some tumor suppressor genes through hypermethylation of the promoter region. In addition to m^5^C, a more complex and dynamic DNA epigenetic regulatory network, including 5-hydroxymethylcytosine (5hmc), 5-formylcytosine (5fC), and 5-carboxycytosine (5caC), has also been identified.^37–39^ Methylation is ubiquitous throughout the genome, and hypomethylation occurs mainly in DNA repeats.^40^ The role of DNA methylation in cancer has been extensively studied. This extensive change in methylation levels can cause gene instability, leading to various tumors, such as hepatocellular carcinoma (HCC),^41,42^ urothelial carcinoma,^43^ and cervical cancer (CC).^44^

With the advancement of technologies, such as RNA sequencing and fluorescence quantification,^45^ the role of RNA methylation in cancer progression has gradually become prominent and an international scientific research hotspot. In addition, research on RNA modification has also made great progress. These modifications were originally thought to be fine-tuned chemical structural features of non-protein-coding RNAs. However, they were now considered dynamically regulated with the identification of a growing number of posttranscriptional regulators. More than 60% of RNA modifications were methylation modifications. Mammalian RNA methylation modifications mainly included N^6^-methyladenosine (m^6^A), N^1^-methyladenosine (m1A), N6,2′-O-dimethyladenosine (m^6^Am), 7-methylguanine (m^7^G), pseudouridine (Ψ), and m^5^C.^46–49^ As the most prevalent RNA methylation modification, m^6^A accounts for 60% of RNA methylation modifications.^50,51^ m^6^A modification has been found in eukaryotic mRNA and long noncoding RNA (lncRNA). It can occur on the adenine of RNA, mRNA, and lncRNA. m^6^A modification plays a significant role in RNA stabilization, localization, transport, splicing, and translation.^52^ m^6^A modification is reversible via the regulation of methyltransferases (“writers”), demethylases (“erasers”), and methylation reading proteins (“readers”).^53,54^ Methyltransferases, such as METTL3/14,^55^ WTAP, and KIAA1429, could catalyze the m^6^A modification of adenosine on mRNA.^53,56^ Demethylases, including FTO and ALKHB5, are used to demethylate bases that have undergone m^6^A modification.^57–59^ The main function of reader proteins is recognized, which is binding to bases with m^6^A modification, thereby activating downstream regulatory pathways, such as RNA degradation and microRNA (miRNA) processing.^60–62^ The abnormality of enzymes involved in m^6^A modification will cause a series of diseases, including tumors, musculoskeletal diseases, and rheumatoid arthritis.^63,64^

RNA m^5^C modification refers to the methylation of the fifth C atom of RNA cytosine.^65,66^ It is widespread in various RNA molecules, including tRNA, rRNA, mRNA, and ncRNA.^67,68^ m^5^C RNA functions by maintaining RNA stability and regulating protein synthesis and translation.^66–68^ m^5^C of tRNA could regulate translation. m^5^C of rRNA could control the quality of ribosome biosynthesis.^68^ m^5^C of mRNA could affect mRNA structure, stability, and translation process.^68^ RNA m^5^C was also regulated by “writers”, “erasers”, and “readers”. m^5^C could be regulated by a series of m^5^C methyltransferases (“writers”), such as NOP2, NSUN2, NSUN3, NSUN4, NSUN5, NSUN6, NSUN7, DNMT1, TRDMT1, DNMT3A, and DNMT3B.^69^ The removal process is catalyzed by TETs. The Aly/REF nuclear export factor (ALYREF) could recognize and bind to m^5^C sites for biological function.^70,71^ The level of m^5^C is closely related to tumorigenesis. NSUN2 promoted gastric cancer (GC) cell proliferation, migration, and invasion by upregulating the m^5^C level.^72^ The m^5^C alteration of PKM2 mRNA improves glucose metabolism in bladder cancer (BLC).^73^

Ψ, one of the trace bases of nucleic acid, is formed by linking the fifth position of uracil and ribose to form pyrimidine nucleoside. The base of Ψ and ribose is not connected by the N–C bond but the C–C bond, which is different from uridine.^74,75^ Ψ, the most abundant modified nucleotide in RNA,^76,77^ is ubiquitous and mainly presented in ncRNA.^78^ Ψ enhances tRNA and rRNA function by stabilizing the RNA structure.^79^ It is directly excreted in the urine, making Ψ a promising biomarker in cancer diagnosis and therapy. In addition, it plays an important role in the regulation of cellular biological functions in tumors.

**Fig. 1:**
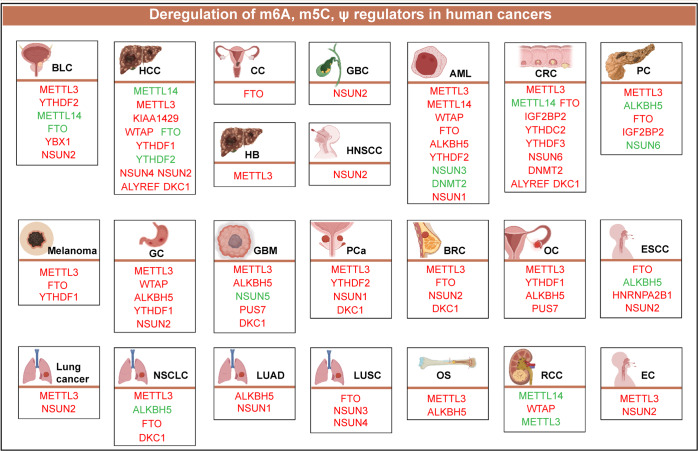
Deregulation of m^6^A, m^5^C, and Ψ regulators in human cancers. Red mains an oncogenic role, while green mains a tumor suppressive role. BLC bladder cancer; HCC hepatocellular carcinoma; CC cervical cancer; GBC gallbladder carcinoma; AML acute myeloid leukemia; CRC colorectal cancer; PC pancreatic cancer; GC gastric cancer; GBM glioblastoma; PCa prostate cancer; BRC breast cancer; OC ovarian cancer; ESCC esophageal squamous cell carcinoma; NSCLC non-small cell lung cancer; LUAD Lung adenocarcinoma; LUSC lung squamous cell carcinoma; OS osteosarcoma; RCC renal cell carcinoma; EC esophageal cancer. Image created with BioRender (https://biorender.com/)

This review detailed and summarized some of the major RNA modifications, including m^6^A, m^5^C, and Ψ. The writers, erasers, and readers related to m^6^A and m^5^C were introduced, and the writers related to Ψ were introduced. Given that the pseudouridylation process is probably irreversible, there were no reports of demethylases. At the same time, no Ψ-related binding protein was found. Second, this review discussed the deposition and function of these major modifications in coding RNAs and ncRNAs. Furthermore, based on the background of RNA modifications, this review summarized and discussed the research status, expression levels, and functions of m^6^A, m^5^C, and Ψ in cancer (Fig. 1). Finally, this review discussed the impact of RNA modifications in cancer treatment and summarized some of the existing small-molecule inhibitors.

## m^6^A writers, erasers, and readers

In 1974, Desrosiers et al.^80^ first discovered m^6^A on the poly(A) tract of mRNA, but due to the lack of technology to detect m^6^A sites in mRNA and the possibility of contamination by rRNA and snRNA, interest in m^6^A was greatly reduced in the late 1970s.^81,82^ However, interest in m^6^A was rekindled in 2012,^83,84^ with the emergence of the next-generation sequencing method MeRIP-Seq as well as genetics and biochemical research.^83,84^ m^6^A mainly appears in the RRACH sequence (where R = A or G, H = A, C, or U), and most sites are located around a stop codon and in long internal exons.^85–87^ m^6^A RNA modification is a dynamic and reversible posttranscriptional process.^88–91^ The level of m^6^A modification of RNA is mainly controlled by so-called “writers”, “erasers”, and “readers”. A writer, mainly including methyltransferase-like 3 (METTL3), METTL14, METTLL16, METTLL5, Wilms tumor 1-associated protein (WTAP), RNA-binding motif protein 15/15B (RBM15/15B), zinc finger CCCH-type containing 13 (ZC3H13), zinc finger CCHC-type-containing 4 (ZCCHC4), and Vir-like m^6^A methyltransferase associated (VIRMA, also known KIAA1429), catalyzes the formation of m^6^A. FTO and alkB homolog 5 (ALKBH5) are two major m^6^A eraser proteins that reversibly remove m^6^A. Readers recognize and bind to the m^6^A site, decode m^6^A methylation and generate a functional signal. These factors mainly include YT521-B homology (YTH) domain-containing protein (including YTHDFs such as YTHDF1/2/3 and YTHDCs such as YTHDC1/2), eukaryotic initiation factor (eIF) 3, IGF2 mRNA-binding protein (IGF2BP) family members (including IGF2BP1/2/3), and heterogeneous nuclear ribonucleoprotein (HNRNP) protein family members (including HNRNPA2B1, HNRNPC, and HNRNPG).

**Fig. 2:**
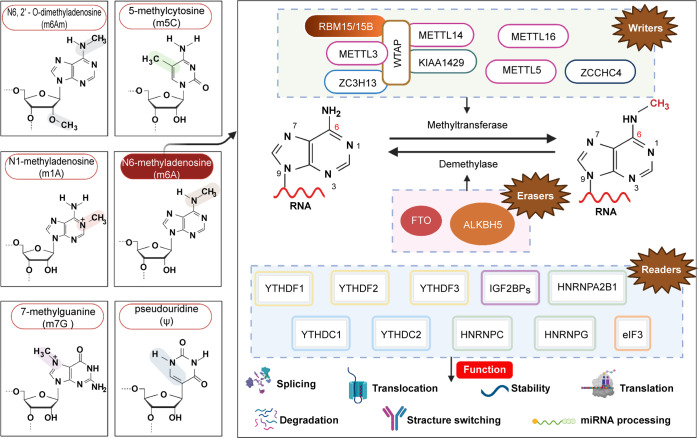
The most common types of RNA modifications and the mechanism of m^6^A regulation. Image created with BioRender (https://biorender.com/)

METTL3 (formerly known as MT-A70) is the most vital component of the m^6^A methyltransferase complex (MTC) and is highly conserved in eukaryotes.^92^ METTL3 was the first identified and most widely known catalytic subunit;^53^ it is an S-adenosylmethionine (SAM)-binding protein that catalyzes the transfer of methyl groups in SAM to adenine bases in RNA.^93–95^ The main function of METTL14 is to stabilize the MTC structure and assist METTL3 in recognizing catalytic substrates.^94,96,97^ WTAP is mainly responsible for recruiting METTL3-METTL14 heterodimers to nuclear speckles and for promoting m^6^A.^98–100^ RBM15/15B, which has no catalytic function, mainly binds to METTL3 and WTAP, directing these two proteins to target RNA sites.^101,102^ Schwartz et al.^97^ and Yue et al.^103^ found that VIRMA preferentially places mRNA methylation modifications near stop codon regions and the 3′-UTR. At the same time, VIRMA recruits the m^6^A complex to the special RNA site.^97,103,104^ ZC3H13 is required for nuclear localization of MTC.^102,103,105^ Moreover, METTL16, METTL5, and ZCCHC4 function alone to add m^6^A to some structural RNAs, such as U6 snRNA, 18S rRNA, and 28S rRNA.^106–111^ FTO and ALKBH5 were the first two proteins discovered to catalyze demethylation of m^6^A, which helps to maintain the dynamic balance of m^6^A modification.^88,112–114^ Imai et al.^115^ first isolated the RNA splicing-related protein YT521 in 1998. Hartmann et al.^116^ then identified the homologous protein YT521-B, and the YT521-B homology (YTH) domain defines a protein family.^117^ YTHDF1 interacts with initiation factors to initiate RNA translation.^118,119^ YTHDF2 mainly regulates the degradation of m^6^A methylated RNA.^120,121^ YTHDF3 acts synergistically with YTHDF1 and YTHDF2 to promote RNA translation and degradation.^122–124^ YTHDC1 facilitates RNA splicing and export,^125,126^ and YTHDC2 enhances translation of a target RNA and reduces target RNA abundance.^127^ By recognizing m^6^A modifications under normal and stress conditions, IGF2BP increases the stability and translation ability of mRNA.^128,129^ Meyer et al.^130^ found that a single 5′-UTR m^6^A results in bypassing of the 5′cap-binding protein and direct binding to eIF3 to promote translation. HNRNPC/G selectively recognizes m^6^A-induced splicing and regulates mRNA abundance.^131,132^ HNRNPA2B1 mediates the processing of primary miRNA (Fig. 2).^133,134^

## Functional consequences of m^6^A in coding RNAs and ncRNAs

m^6^A modification has been a hot research topic in recent years. m^6^A is a common modification that regulates gene expression in eukaryotes,^135,136^ and the discovery of FTO reveals the reversibility and dynamic balance of m^6^A.^88^ Constantly emerging studies showed that m^6^A is present on both mRNA and ncRNAs.^137,138^ This review summarized the functional consequences of m^6^A in mRNA and ncRNAs (mainly including miRNA, lncRNA, and circRNA).

### Role of m^6^A in mRNA

mRNA is the template for protein biosynthesis.^139^ The mature mRNA of eukaryotic cells has a cap structure of trans-7-methylguanine triphosphate nucleoside at the 5′-end and a poly(A)­tail structure at the 3′-end. The primary product of mRNA is known as heterologous RNA (hnRNA). After a series of posttranscriptional modifications, hnRNA is spliced into mature mRNA that is finally transported to the cytoplasm. During translocation of hnRNA into the cytoplasm, introns are spliced out and exons are joined together. After further capping and polyadenylation modifications, hnRNA becomes mature mRNA.^140,141^

m^6^A is the most abundant chemical modification in mRNA and plays an important role in multiple processes, such as cell differentiation and tissue development.^122^ m^6^A affects virtually every stage of mRNA metabolism, from processing in the nucleus to transport to translation and degradation in the cytoplasm.^142^ As described previously, the “readers” recognize and bind to m^6^A sites, decode m^6^A methylation, and generate functional signals. The role of m^6^A in mRNA is mainly recognized and regulated by “readers.” m^6^A “readers” have two different functional modes: direct and indirect reading. Direct reading refers to the selective binding of m^6^A to RNA-binding proteins with diverse cellular functions.^142^ If m^6^A modifications slightly reduce the A: U base pairing energy, this difference may alter the RNA secondary structure and thus alter RNA and protein interactions. It can cause indirect reading.^143,144^ The specific role of “readers” has already been elaborated on in the previous section. In general, m^6^A can enhance the nuclear processing, splicing, and export of mRNA. It can promote mRNA translation and affect mRNA degradation. There is a synergy among the YTHDF family. YTHDF3 affects the translation and decay of methylated mRNAs by cooperating with YTHDF1 and YTHDF2. Although both YTHDF1 and YTHDF3 contribute to the translation of their target mRNAs, they also collectively affect the segmentation of YTHDF2 by methylated transcripts to accelerate mRNA degradation.^122^ In addition, studies also reported that the demethylation activity of ALKBH5 significantly affects mRNA export and RNA metabolism and the assembly of mRNA processing factors in nuclear speckles.^145^

### Role of m^6^A in miRNA

miRNAs are ncRNAs with ~22 nucleotides.^146–148^ Under the guidance of the RNA-induced silencing complex, miRNA and the 3′-untranslated region (3′-UTR) of the target gene pair complementarily regulate gene expression, resulting in mRNA degradation or translational inhibition at the posttranscriptional level.^149–151^ The binding of miRNAs and mRNA regulates ~60% of coding genes.^152^ The maturation process of miRNA mainly includes three steps.^153,154^ First, miRNA is transcribed into a long and capped precursor primary miRNA (pri-miRNA) by RNA polymerase II in the nucleus.^155^ Second, with the assistance of Drosha ribonuclease III and DiGeorge syndrome key region gene 8 (DGCR8), pri-miRNA is converted to pre-miRNA.^156,157^ Third, pre-miRNA is exported to the cytoplasm, where it is cleaved by the RNase III endonuclease Dicer to produce mature miRNA.^158–160^ Studies showed that m^6^A modification and its regulatory factors are widely involved in miRNA processing and maturation.^161–164^ Studies revealed the role of METTL3 in promoting miRNA maturation.^163,165,166^ For example, Liang et al.^167^ revealed that METTL3 promoted the binding of DGCR8 to miR-20a-5p and m^6^A modification, thereby increasing miR-20a-5p expression and inhibiting NFIC transcription. m^6^A induces the processing of pri-miR-17-92 in an m^6^A/DGCR8-dependent manner. The m^6^A modification that mediates this process occurs at the A879 site of pri-miR-17-92.^168^ METTL14 can also affect miRNA expression by regulating pri-miRNA processing and maturation.^169,170^ Lin et al.^171^ found that DCA reduces miR-92b-3p expression through m^6^A-relied posttranscriptional modification by promoting the dissolution of the METTL3-METTL14-WTAP complex. In addition to the role of writers in miRNA processing, erasers affect miRNA maturation. Chen et al.^172^ discussed the potential role of the ALKBH5/miR-194-2/RAI1 axis in esophageal squamous cell carcinoma (ESCC) treatment. ALKBH5 mainly demethylates pri-miR-194-2 and inhibits miR-194-2 biogenesis in an m^6^A/DGCR8-dependent manner.

Interestingly, miRNAs also affect the expression of m^6^A-modified proteins, modulating the level of downstream target genes.^173,174^ For example, METTL3 is a direct target of miR-186 in hepatoblastoma (HB) cells.^175^ miR-4429 inhibits SEC62 stabilization caused by m^6^A by targeting METTL3 in GC to suppress GC cell proliferation.^176^ Yue et al.^177^ found that miR-96 downregulates AMPKα2, increasing FTO expression. FTO in turn upregulates MYC expression by blocking m^6^A modification of MYC. This mechanism is involved in the pro-proliferation and antiapoptotic effects of miR-96 in colorectal cancer (CRC) cells. Xue et al.^178^ highlighted a positive feedback loop between ALKBH5 and miR-193a-3p. miR-193-3p targets ALKBH5 to inhibit its expression; ALKBH5 in turn inhibits miR-193a-3p expression. This positive feedback loop further promotes ESCC cell growth and metastasis.

### Role of m^6^A in lncRNA

LncRNAs are non-protein-coding RNAs with >200 nucleotides,^179,180^ which regulate gene expression at the transcriptional or posttranscriptional level and are involved in physiological and pathological processes.^181,182^ m^6^A is the most abundant RNA modification in mammalian mRNA and lncRNA, with an average of three to five sites in each transcript.^183^ lncRNA X-inactive specific transcripts (XISTs) mediate the transcriptional silencing of genes on the X chromosome. XIST was highly methylated. METTL3 knockdown disrupted XIST-mediated gene silencing. YTHDC1 preferentially recognized m^6^A residues on XIST that were required for XIST to function.^101,184^ In addition, METTL14 inhibits the growth and metastasis of CRC by downregulating the carcinogenic lncRNA XIST.^185^ Hu and Ji^186^ found that METTL14-mediated m^6^A modification results in LINC01320 upregulation. Upregulated LINC01320 promotes GC cell invasion by regulating the miR-495-5p/RAB19 axis. METTL3-mediated m^6^A modification resulted in the downregulation of lncRNA MEG3; downregulated MEG3 regulates BTG2 expression through miR-544b.^187^ Erasers and readers also regulate lncRNA expression. Zhang et al.^188^ revealed that ALKBH5 promotes GC invasion and metastasis by reducing lncRNA NEAT1 methylation. ALKBH5 inhibits the motility of pancreatic cancer by reducing lncRNA KCNK15-AS1 methylation.^189^ Cui et al.^190^ found that FTO reduces m^6^A methylation of the LINC00022 transcript, resulting in the attenuation of LINC00022 inhibition by YTHDF2. Ni et al.^191^ pointed out that YTHDF3 is not only a new target of YAP but also has a key role in the YAP pathway by promoting the degradation of m^6^A-modified lncRNA GAS5.

### Role of m^6^A in circRNA

circRNAs comprise a special class of ncRNA molecules that form a ring structure through typical 5′-3′-phosphodiester bonds;^192–194^ no polyadenylated tail at the 3′-end and no cap structure at the 5′-end are present.^195^ Compared to linear ncRNA, circRNA has a high degree of stability due to its covalent closed-loop structure.^196,197^ According to reports, circRNAs are widely involved in the occurrence and development of cancer.^198,199^ More importantly, circRNAs can be detected in the blood, rendering these molecules ideal biomarkers for cancer diagnosis and prognosis. Recently, m^6^A modifications of circRNAs have been widespread.^200–202^ m^6^A can regulate circRNA translation. The initiation factor eIF4G2 and the m^6^A reader YTHDF drive translation initiation, and METTL3/14 further enhance translation.^203,204^ As mentioned above, circRNAs are more stable than other ncRNAs due to their closed-loop structure and are not easily degraded. Therefore, studies explored whether m^6^A modification affects circRNA degradation. The answer is yes. They found that m^6^A-modified circRNAs were also endoribonuclease-cleaved via the YTHDF2-HRSP12-RNase P/MRP axis.^205^ Guo et al.^206^ revealed that circ3823 degradation might be regulated by YTHDF3 and ALKBH5. In addition, m^6^A modification of circRNAs also suppressed innate immunity.^207,208^ According to Chen et al.,^209^ circNSUN2 is exported from the nucleus to the cytoplasm by YTHDC1 in an m^6^A methylation-dependent manner. Chen et al.^210^ also found that METTL3 induces circ1662 expression, accelerating YAP1 nuclear transport. Liu et al.^211^ found that circDLC1 is a new downstream effector of m^6^A modification mediated by KIAA1429. Xu et al.^212^ reported that m^6^A-modified circRNA ORE maintains sorafenib resistance in HCC patients by regulating the β-catenin signaling pathway. In another HCC study, the HBx protein upregulated METTL3 expression and increased the m^6^A modification of circARL3. YTHDC1 interacts with circ-ARL3 m^6^A modification to promote its reverse splicing and biogenesis.^213^

## Role of m^6^A in human cancers

### m^6^A and digestive system neoplasms

Digestive system neoplasms mainly include esophageal cancer (EC), GC, liver cancer, CRC, small bowel cancer, pancreatic cancer (PC), and gallbladder cancer. Digestive system cancer is a huge health burden worldwide, accounting for approximately 35% of global cancer-related mortality.^214–216^ Despite significant progress in digestive system cancer treatment regarding conventional surgical resection, neoadjuvant chemotherapy, radiotherapy, and immunotherapy, the overall survival rate remains very low, and morbidity and mortality rates are still rising.^217–219^ Therefore, new technologies are needed to detect early-stage digestive system cancer and to find new treatment methods and effective targets. Recently, there have been many studies on m^6^A expression with regard to the prognosis of digestive system cancer.^220–225^ Li et al.^226^ discussed the expression pattern and prognostic significance of m^6^A-related genes in ESCC, reporting that YTHDF1, HNRNPC, ZC3H13, YTHDC2, and METTL14 are dysregulated in ESCC. In addition, they found that patients with high levels of ALKBH5 have better overall survival but that patients with HNRNPC and WTAP overexpression have worse overall survival. Guan et al.^227^ conducted a similar study on GC: compared with normal tissues, most m^6^A-related genes were upregulated at both the protein and mRNA levels in GC. The expression level of m^6^A-related genes is also closely related to clinicopathological characteristics such as age, TNM stage, and race. High expression of WTAP and FTO indicates a poor prognosis for GC patients. Liu et al.^228^ analyzed RNA-seq FPKM data and matched clinical data for 331 CRC samples, finding only ALKBH5 and METTL14 to be downregulated in tumors. YTHDF1 and HNRNPC can be used as prognostic factors for CRC, with potential value in CRC treatment. Similar prediction models have been carried out in HCC and PC.^129,229–234^ Overall, the development of various sequencing technologies has stimulated interest in m^6^A and cancer research, laying a foundation for cancer markers and the development of new cancer treatment methods.

**Fig. 3:**
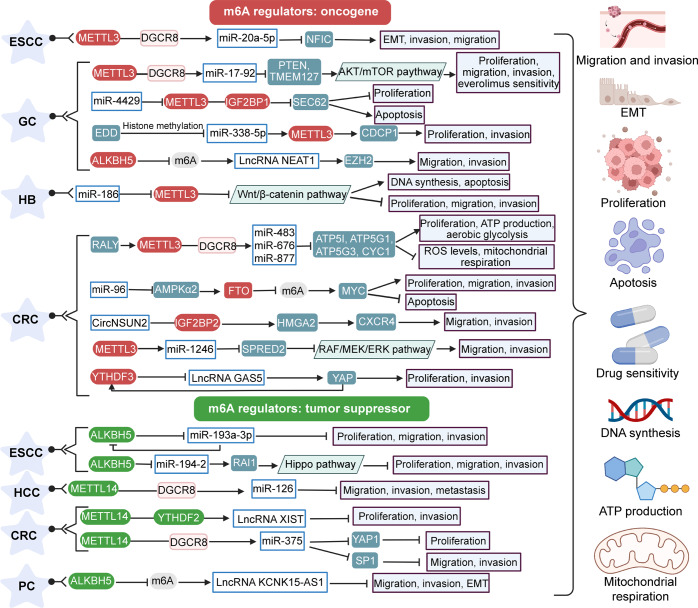
The role of m^6^A in digestive system neoplasms. m^6^A can not only promote cancer progression, but it also plays a role in inhibiting progression. This figure mainly proposes how writers, erasers, and readers participate in the regulation of various genes and pathways in cancer progression. ESCC esophageal squamous cell carcinoma; GC gastric cancer; HB hepatoblastoma; CRC colorectal cancer; HCC hepatocellular carcinoma; PC pancreatic cancer. Image created with BioRender (https://biorender.com/)

METTL3 and METTL14 are the two most common m^6^A methyltransferases. Research shows that METTL3 is mainly expressed as an oncogene in digestive system cancer but that METTL14 plays a role in inhibiting cancer. Indeed, METTL3 is upregulated in a variety of digestive system cancers. Highly expressed METTL3 is closely linked to lymph node involvement, distant metastasis, stage, and microvascular invasion, among other factors. It is also involved in multiple activities, such as proliferation, migration, invasion, and apoptosis.^165,167,168,173,176,235–239^ Sun et al.^161^ found that downregulation of METTL3 significantly inhibits the growth of CRC cells. Additionally, overexpression of miR-877 rescues the effects of METTL3 deletion on mitochondrial respiration and aerobic glycolysis. METTL3 can also promote DNA synthesis in HB. METTL3 is overexpressed in HB tissues and cell lines, and high levels are associated with the poor prognosis of HB patients.^175^ METTL14 is downregulated in CRC and HCC and mainly inhibits the occurrence and outcome of cancer by suppressing the proliferation and migration of cancer cells. Low expression of METTL14 is also greatly associated with clinicopathological characteristics such as TNM stage, differentiation, tumor encapsulation, tumor microsatellite, and microvascular invasion.^169,170,185,240^ FTO and ALKBH5 are demethylases that maintain the dynamic balance of m^6^A modification. According to Yue et al.,^177^ FTO expression is increased in CRC. FTO induces the proliferation and invasion of CRC cells by upregulating the c-Myc proto-oncogene (MYC) and inhibits apoptosis. FTO also plays a cancer-promoting role in PC.^241^ Zhang et al.^188^ studied the role of ALKBH5 in GC. Both western blot and RT–qPCR results indicated that ALKBH5 is overexpressed in GC. ALKBH5 mainly upregulates EZH2 by demethylating lncRNA NEAT1, promoting GC cell invasion and migration. The tumor-suppressor effect of ALKBH5 has been shown in ESCC and PC.^172,178,189^ The reason for this contradiction may be the different targets of ALKBH5. To date, most studies on m^6^A in cancer have been based on the m^6^A modification itself rather than on the underlying mechanism of reader proteins. In fact, the role of various m^6^A reader proteins in cancer remains largely unexplored.^119,242^ Table 1 summarizes the role of m^6^A reader in cancer reported by some current studies.^243–246^ For example, Chen et al.^209^ found that IGF2BP2 enhanced the stabilianticodon stem-loopty of HMGA2, thereby promoting the metastasis of CRC cells and exerting a cancer-promoting effect. YTHDF3 is also expressed as an oncogene in CRC,^191^ and Yang et al.^185^ described a ‘METTL14-YTHDF2-lncRNA’ regulatory axis in CRC cells (Fig. 3).

**Table 1:** RNA modifying regulators of m^6^A in human cancers

	Cancer type	Category	m^6^A regulators	Expression (tumor vs. normal)	Property	Prognostic implication of m^6^A regulators overexpression	Functional role	Molecular mechanism	Related genes	Refs.
Digestive System Neoplasms	Esophageal cancer	Writer	METTL3	Upregulation	Oncogene	/	Proliferation↑, migration↑, invasion↑, apoptosis ↓	Activates Wnt3/β-catenin and AKT signaling pathways	/	235
		Writer	METTL3	Upregulation	Oncogene	/	EMT↑, invasion↑, migration ↑	Promotes m^6^A modification and DGCR8 binding to miR-20a-5p	DGCR8, miR-20a-5p, NFIC	167
	Esophageal squamous cell carcinoma	Eraser	FTO	Upregulation	Oncogene	Poor	Proliferation↑, migration ↑	Upregulates the expression of MMP13	MMP13	223
		Eraser	ALKBH5	Downregulation	Tumor suppressor	/	Proliferation↓, migration↓, invasion ↓	Positive feedback exists between miR-193a-3p and ALKBH5	DGCR8, miR-193a-3p	178
		Eraser	ALKBH5	Downregulation	Tumor suppressor	Poor	Proliferation↓, migration↓, invasion↓, colony formation ↓	Inhibits m^6^A/DGCR8-dependent miRNA biogenesis and releases RAI1 expression	DGCR8, miR-194-2, RAI1	172
		Reader	HNRNPA2B1	Upregulation	Oncogene	Poor	Proliferation↑, migration↑, invasion↑, fatty acid synthesis ↑	Upregulates the expression of ACLY and ACC1	ACLY, ACC1	243
	Gastric cancer	Writer	METTL3	Upregulation	Oncogene	Poor	Proliferation↑, colony formation↑, migration↑, invasion↑, peritoneal metastasis↑, sensitivity to everolimus ↑	Promotes pri-miR-17-92 processing through an m^6^A/DGCR8-dependent mechanism	DGCR8, miR-17-92, PTEN, TMEM127	168
		Writer	METTL3	Upregulation	Oncogene	/	Proliferation↑, apoptosis ↓	Mediated m^6^A modification promotes stabilization of SEC62 mRNA by IGF2BP1	miR-4429, SEC62	176
		Writer	METTL3	Upregulation	Oncogene	/	Proliferation↑, invasion ↑	Inceases CDCP1 expression	EED, miR-338-5p, CDCP1	173
		Writer	METTL3	Upregulation	Oncogene	/	Proliferation↑, migration↑, invasion↑, apoptosis ↓	Enhances MYC m^6^A methylation and translation	HBXIP, MYC	239
		Writer	WTAP	Upregulation	/	Poor	T cell infiltration ↓	/	/	224
		Eraser	ALKBH5	Upregulation	Oncogene	/	Migration↑, invasion ↑	Demethylates NEAT1 and upregulates its expression	lncRNA NEAT1, EZH2	188
		Reader	YTHDF1	Upregulation	Oncogene	Poor	Proliferation↑, metastasis ↑	Promotes translation of FZD7 in an m^6^A-dependent manner	FZD7	244
	Hepatocellular carcinoma	Writer	METTL14	Downregulation	Tumor suppressor	Favorable	Migration↓, invasion↓, metastasis ↓	Promotes pri-miR-126 processing through an m^6^A/DGCR8-dependent mechanism	DGCR8, miR-126	170
		Writer	METTL3	Upregulation	Oncogene	Poor	Proliferation↑, migration↑, invasion↑, adipogenesis ↑	Promotes expression of LINC00958 in an m^6^A-dependent manner	LINC00958, miR-3619-5p, HDGF	232
		Writer	KIAA1429	Upregulation	Oncogene	Poor	Proliferation↑, metastasis ↑	Induces GATA3 pre-mRNA m^6^A methylation, promoting its degradation	LncRNA GATA3-AS, GATA3	233
		Writer	WTAP	Upregulation	Oncogene	Poor	Proliferation↑, migration ↑	Inhibits ETS1 expression in an m^6^A-HuR-mediated manner	ETS1	234
		Eraser	FTO	Downregulation	Tumor suppressor	Favorable	/	/	/	222
		Reader	YTHDF1	Upregulation	Oncogene	Poor	/	/	/	225
		Reader	YTHDF2	Downregulation	Tumor suppressor	Favorable	Growth↓, vascular density and permeability↓, inflammation ↓	Degrades IL11 and SERPINE2 mRNA	HIF-2α, IL11, SERPINE2	245
	Hepatoblastoma	Writer	METTL3	Upregulation	Oncogene	Poor	Proliferation↑, colony formation↑, migration↑, invasion↑, DNA synthesis↑, apoptosis ↓	miR-186 negatively regulates METTL3	miR-186	175
	Colorectal cancer	Writer	METTL3	Upregulation	Oncogene	Poor	Migration↑, invasion ↑	Methylates pri-miR-1246 to promote its maturation	miR-1246, SPRED2	165
		Writer	METTL3	/	Oncogene	/	Proliferation↑, ATP production↑, aerobic glycolysis↑, ROS levels↓, mitochondrial respiration ↓	m^6^A modification promotes miRNA processing	RALY, miR-483, miR-676, miR-877	161
		Writer	METTL3	Upregulation	Oncogene	Poor	Proliferation↑, colony formation↑, apoptosis ↓	Promotes GLUT1 protein expression in an m^6^A-dependent manner	GLUT1	236
		Writer	METTL3	Upregulation	Oncogene	Poor	Tumor self-renewal↑, cell invasion↑, chemoresistance ↑	Promotes SOX2 expression in an m^6^A-IGF2BP2-dependent manner	SOX2	237
		Writer	METTL14	Downregulation	Tumor suppressor	Favorable	Migration↓, invasion↓, metastasis ↓	Promotes SOX4 expression in an m^6^A-YTHDF2-dependent manner	SOX4	240
		Writer	METTL14	Downregulation	Tumor suppressor	Favorable	Proliferation↓, invasion ↓	Downregulation of XIST via an m^6^A-YTHDF2-dependent pathway	LncRNA XIST	185
		Writer	METTL14	Downregulation	Tumor suppressor	Favorable	Proliferation↓, migration↓, invasion ↓	Promotes pri-miR-375 processing through an m^6^A/DGCR8-dependent mechanism	miR-375, YAP1, SP1	169
		Eraser	FTO	Upregulation	Oncogene	/	Proliferation↑, migration↑, invasion↑, apoptosis ↓	Demethylates MYC and upregulates its expression	miR-96, AMPKα2-, MYC	177
		Reader	IGF2BP2	/	Oncogene	/	Migration↑, invasion ↑	Enhances stability of HMGA2 in an m^6^A modification-independent manner	HMGA2, CXCR4	209
		Reader	YTHDC2	Upregulation	Oncogene	/	Metastasis ↑	Increases translation of HIF-1α mRNA	HIF-1α	246
		Reader	YTHDF3	Upregulation	Oncogene	/	Proliferation↑, invasion ↑	Degradation of GAS5 by m^6^A modification	LncRNA GAS5, YAP	191
	Pancreatic cancer	Writer	METTL3	Upregulation	Oncogene	/	Proliferation↑, migration↑, invasion ↑	/	/	238
		Eraser	ALKBH5	Downregulation	Tumor suppressor	/	Migration↓, invasion↓, EMT↓	Demethylates lncRNA KCNK15-AS1	LncRNA KCNK15-AS1	189
		Eraser	FTO	Upregulation	Oncogene	/	Proliferation ↑	Regulates the m^6^A expression level of PANC-1 mRNA	PANC-1	241
		Reader	IGF2BP2	Upregulation	Oncogene	Poor	Proliferation↑, stem cell-like properties ↑	Regulates DANCR stability	LncRNA DANCR	129
Respiratory system neoplasms	Lung cancer	Writer	METTL3	Upregulation	Oncogene	/	EMT↑	Reduces m^6^A levels on JUNB mRNA	JUNB	250
		Writer	METTL3	/	/	/	Brain metastases ↑	Promotes splicing of pre-miR-143-3p	miR-143-3p, VASH1	251
		Reader	YTHDF2	Upregulation	Oncogene	/	Proliferation ↑	Promotes 6PGD mRNA translation	6PGD	249
	Non-small cell lung cancer	Writer	METTL3	Upregulation	Oncogene	Poor	Proliferation↑, migration↑, invasion↑, drug resistance ↑	Enhanced YAP mRNA translation	YAP, MALAT1, miR-1914-3p	254
		Eraser	ALKBH5	Downregulation	Tumor suppressor	Favorable	Proliferation↓, migration↓, invasion↓, EMT↓	Reduces m^6^A levels on YAP pre-mRNA	YAP, miR-107, LATS2	256
		Eraser	FTO	Upregulation	Oncogene	/	Proliferation ↑	Reduces the m^6^A level of USP7 mRNA	USP7	252
	Lung adenocarcinoma	Eraser	ALKBH5	Upregulation	Oncogene	/	Proliferation↑, invasion ↑	Reduces the m^6^A level of FOXM1 mRNA	FOXM1	255
	Lung squamous cell carcinoma	Eraser	FTO	Upregulation	Oncogene	Poor	Proliferation↑, invasion↑, apoptosis ↓	Reduces the m^6^A level of MZF1 mRNA	MZF1	253
Urinary system neoplasms	Bladder cancer	Writer	METTL3	Upregulation	Oncogene	/	Proliferation↑, invasion↑, apoptosis ↓	A multi-level regulatory network exists downstream of METTL3	AFF4, IKBKB, REL, MYC	260
		Writer	METTL3	Upregulation	Oncogene	/	Proliferation↑, migration↑, invasion ↑	Promotes CDCP1 mRNA modification and translation	CDCP1	261
		Writer	METTL3	Upregulation	Oncogene	Poor	Proliferation ↑	Accelerates maturation of pri-miR-221/222	miR-221/222	264
		Writer	METTL3	Upregulation	Oncogene	/	Adhesion↑, migration↑, invasion ↑	Promotes translation of ITGA6 mRNA	ITGA6	262
		Writer	METTL3	Upregulation	Oncogene	/	Proliferation↑, migration ↑	Degrades SETD7 and KLF4 mRNA	SETD7, KLF4	263
		Reader	YTHDF2	Upregulation	Oncogene	/	Migration ↑	Degrades SETD7 and KLF4 mRNA	SETD7, KLF4	263
		Writer	METTL14	Downregulation	Tumor suppressor	/	Proliferation↓, self-renewal↓, metastasis↓, tumor initiating capacity ↓	Notch1 m^6^A modification inhibits its RNA stability	Notch1	58
		Eraser	FTO	Downregulation	Tumor suppressor	/	Proliferation↓, migration ↓	/	/	265
	Renal cell carcinoma	Writer	METTL14	Downregulation	Tumor suppressor	Favorable	Migration↓, invasion ↓	Depletes P2RX6 protein levels by m^6^A modification	P2RX6	277
		Writer	WTAP	Upregulation	Oncogene	Poor	Proliferation ↑	Stabilizes CDK2 transcripts to enhance its expression	CDK2	278
		Writer	METTL3	Downregulation	Tumor suppressor	Favorable	Proliferation↓, migration↓, invasion↓, cell cycle arrest in G1 phase ↑	Involves PI3K/Akt/mTOR signaling pathway	/	276
	Prostate cancer	Writer	METTL3	Upregulation	Oncogene	Poor	Adhesion↑, migration ↑	Enhances mRNA stability of ITGB1 by m^6^A modification	ITGB1	282
		Writer	METTL3	Upregulation	Oncogene	/	Proliferation↑, migration↑, invasion↑, apoptosis ↓	Increases the expression of GLI1 by m^6^A modification	GLI1	283
		Reader	YTHDF2	Upregulation	Oncogene	/	Proliferation↑, migration ↑	miR-493-3p targets YTHDF2	miR-493-3p	284
Female reproductive system neoplasms	Ovarian cancer	Writer	METTL3	Upregulation	Oncogene	Poor	Proliferation↑, migration↑, invasion↑, EMT↑	Promotes AXL translation	AXL	288
		Writer	METTL3	Upregulation	Oncogene	/	Proliferation↑, migration↑, invasion↑, apoptosis ↓	Mediates by reducing activation of the AKT signaling pathway	/	289
		Reader	YTHDF1	Upregulation	Oncogene	/	Proliferation↑, migration↑, invasion ↑	Enhances EIF3C translation by m^6^A modification	EIF3C	290
		Eraser	ALKBH5	Upregulation	Oncogene	Poor	Proliferation↑, migration↑, autophagy ↓	Enhances BCL-2 mRNA stability by catalyzing m^6^A demethylation	BCL-2	291
		Eraser	ALKBH5	Upregulation (down‐regulated in OC cell lines)	Oncogene	/	Proliferation↑, apoptosis ↓	TLR4 upregulates ALKBH5 expression by activating NF-κB signaling pathway	TLR4	292
	Cervical cancer	Eraser	FTO	Upregulation	Oncogene	/	Proliferation↑, migration ↑	Control of m^6^A modification of E2F1 and Myc transcripts	E2F1, Myc	298
		Eraser	FTO	Upregulation	Oncogene	Poor	Drug resistance ↑	Reduces m^6^A of β-catenin mRNA, thereby positively regulating β-catenin expression	/	299
Nervous system neoplasms	Glioblastoma	Writer	METTL3	Upregulation	Oncogene	Poor	Proliferation↑, migration ↑	Mediates nonsense-mediated decay of SRSFs mRNA	SRSF	307
		Writer	METTL3	Upregulation	Oncogene	/	Radioresistance ↑	Enhances SOX2 mRNA stability by m^6^A modification	SOX2	306
		Eraser	ALKBH5	Upregulation	Oncogene	Poor	GSCs proliferation ↑	Demethylates FOXM1 nascent transcripts, resulting in enhanced FOXM1 expression.	FOXM1, lncRNA FOXM1-AS	305
Hematological neoplasms	Acute myeloid leukemia	Writer	METTL3	Upregulation	Oncogene	/	Proliferation↑, myeloid differentiation↑, apoptosis ↓	Directly controls the expression of c-MYC, BCL-2, and PTEN	c-MYC, BCL-2, PTEN	317
		Writer	METTL14	Upregulation	Oncogene	/	Proliferation ↑	Regulation of its mRNA targets through m^6^A modification	MYB, MYC SPI1	321
		Writer	WTAP	Upregulation	Oncogene	/	Proliferation↑, apoptosis ↓	/	/	315
		Eraser	FTO	Upregulation	Oncogene	/	Proliferation↑, apoptosis ↓	Regulates the expression of targets such as ASB2 and RARA by reducing m^6^A levels	ASB2, RARA	318
		Eraser	ALKBH5	Upregulation	Oncogene	Poor	Proliferation↑, self-renewal of LSCs/LICs ↑	Post-transcriptional regulation of its key targets, such as TACC3	TACC3	319
		Reader	YTHDF2	Upregulation	Oncogene	/	Proliferation↑, apoptosis ↓	/	/	316
Tumors of other systems	Breast cancer	Writer	METTL3	Upregulation	Oncogene	Poor	Proliferation↑, apoptosis ↓	Promotes Bcl-2 translation by m^6^A modification	Bcl-2	322
		Writer	METTL3	Upregulation	Oncogene	Poor	Proliferation↑, apoptosis ↓	Positive feedback loop for HBXIP/let-7g/METTL3/HBXIP	HBXIP, miR-let-7g	323
		Eraser	FTO	Upregulation	Oncogene	Poor	Proliferation↑, colony formation↑, metastasis ↑	Mediates m^6^A demethylation of BNIP3 mRNA, inducing its degradation	BNIP3	324
	Melanoma	Writer	METTL3	Upregulation	Oncogene	/	Proliferation↑, migration↑, invasion ↑	Post-transcriptional regulation of c-Met expression	c-Met	325
		Writer	METTL3	Upregulation	Oncogene	/	Colony formation↑, invasion ↑	Promotes accumulation of MMP2 and N-cadherin	MMP2	326
		Eraser	FTO	Upregulation	Oncogene	/	Drug resistance ↑	/	/	327
		Reader	YTHDF1	/	Oncogene	/	Proliferation↑, migration ↑	Promotes translation of methylated HINT2 mRNA	HINT2	328
	Osteosarcoma	Writer	METTL3	Upregulation	Oncogene	/	Proliferation↑, migration↑, invasion ↑	Regulates the m^6^A level of LEF1 and activates the Wnt/β-catenin signaling pathway	LEF1	329
		Eraser	ALKBH5	Upregulation	Oncogene	/	Proliferation ↑	Reduces m^6^A modification of PVT1, mediating PVT1 upregulation	PVT1	330

### m^6^A and respiratory system neoplasms

Among respiratory tumors, current research is mainly concerned with m^6^A and lung cancer. Lung cancer is a common malignant tumor, mainly divided into small cell lung cancer (SCLC) and non-SCLC (NSCLC). Among them, NSCLC accounts for about 85% of lung cancer cases.^247^ NSCLC can be further divided into two subtypes: lung adenocarcinoma (LUAD) and lung SCC (LUSC), accounting for 50–60% and 30% of the total cases, respectively. Studies found that the m^6^A modification of RNA is widely involved in the occurrence and development of lung cancer.^248^ It was concluded that m^6^A regulatory proteins, whether “writers”, “erasers”, or “readers”, play a role in lung cancer. In most cases, these regulatory proteins are mainly upregulated in lung cancer and play a cancer-promoting role.^249–254^ ALKBH5 increases FOXM1 expression by downregulating m^6^A modification on FOXM1 mRNA, ultimately promoting LUAD cell proliferation and invasion in an intermittent hypoxic environment.^255^ However, Jin et al.^256^ indicated that ALKBH5 expression is reduced in NSCLC, and high ALKBH5 expression predicts a good clinical prognosis. Molecularly, ALKBH5 reduced YAP activity by regulating the miR-107/LATS2 axis in a HuR-dependent manner, further inhibiting tumor growth and metastasis in vivo. Although there is no direct contradiction between the studies, more research is worthwhile to further verify the role and mechanism of m^6^A modification and its related regulators in lung cancer.

### m^6^A and urinary system neoplasms

#### Bladder cancer (BLC)

BLC is one of the most common malignant tumors of the urinary tract, especially in developed countries. Despite recent advances in clinical treatment, BLC remains a major cause of cancer-related morbidity and mortality due to its high heterogeneity and recurrence rate.^257–259^ Numerous studies documented the importance of m^6^A modifications in biological processes and disease pathogenesis, including in BLC. Most studies revealed that METTL3, as an m^6^A-modified “writer,” can promote m^6^A levels of targeted mRNAs and translational levels and possibly plays a role in promoting mRNA stability.^260–262^ In addition, Xie et al.^263^ found that METTL3 and YTHDF2 synergistically degrade mRNAs of the tumor suppressors SETD7 and KLF4, thereby promoting BLC development. In another study, METTL3 may positively regulate the processing of pri-miR221/222 into mature miRNA by interacting with DGCR8, thereby playing an oncogenic role in BLC.^264^ Of course, other studies showed that METTL14 and FTO are under-expressed in BLC and mainly play a tumor suppressor role.^58,265^

#### Renal cell carcinoma (RCC)

RCC accounts for about 90% of renal cancers and is the most lethal of urogenital malignancies. Clear cell RCC (ccRCC) is the most common histological subtype.^266,267^ About 400,000 patients worldwide are diagnosed with kidney cancer each year, and the mortality rate is very high.^268,269^ Because RCC is resistant to chemotherapy and radiotherapy, surgery remains the most effective treatment for patients with RCC.^270–273^ However, patients with metastatic RCC may not have the opportunity for surgery and have a poor prognosis.^274^ Therefore, new targeted therapy is urgently needed to improve the survival and prognosis of RCC patients. The identification of m^6^A RNA methylation regulators has led to new insights into the potential therapeutic roles of m^6^A modifications in gene expression regulation and cancer. Genetic alterations in m^6^A regulators in ccRCC were identified for the first time, and a significant association between these alterations and worse clinical features was found.^275^ Unlike other cancers, this study showed that METTL3 was upregulated in RCC, and METTL3-negative expression was associated with larger tumor size (*P* = 0.010) and higher histological grade (*P* = 0.021). Further research revealed that METTL3 might inhibit RCC cell proliferation, migration, and invasion through the phosphatidylinositol 3-kinase (PI3K)-Akt-mammalian target of rapamycin (mTOR) pathway and play a tumor suppressor role.^276^ Another major m^6^A methyltransferase, METTL14, also inhibits RCC progression.^277^ Interestingly, however, WATP is upregulated in RCC and upregulates CDK2 expression by stabilizing its transcripts, promoting RCC tumorigenesis. WATP is also a methyltransferase and is mainly responsible for the recruitment of METTL3 and METTL14. However, whether this cancer-promoting effect of WATP is directly related to m^6^A modification was not investigated.^278^ More in-depth studies are needed to further explore the mechanism of action of m^6^A and RCC.

#### Prostate cancer (PCa)

PCa is also a common cancer of the urinary system and is the second leading cause of cancer-related deaths in men worldwide.^267,279,280^ Surgery, chemoradiotherapy, and hormone therapy are effective treatments for PCa, but the recurrence rate of patients is still high, and the mortality rate has not decreased.^281^ Therefore, further elucidation of the profound mechanisms involved in PCa progression is of great interest. Studies revealed the cancer-promoting role of m^6^A in PCa. METTL3 further affects downstream mechanisms mainly by affecting target mRNA stability and expression levels, ultimately promoting PCa cell proliferation, migration, invasion, and adhesion and inhibiting apoptosis.^282,283^ YTHDF2, a “reader” for m^6^A, is upregulated in PCa. Furthermore, miR-493-3p could target YTHDF2 and inhibit YTHDF2 expression, thereby reversing the cancer-promoting effect of YTHDF2.^284^

### m^6^A and female reproductive system neoplasms

#### Ovarian cancer (OC)

OC is one of the most common gynecological tumors.^285^ Patients with advanced OC have a poor prognosis and high recurrence rates.^286,287^ There is an urgent need to identify and validate specific biomarkers and therapeutic targets for OC. Likewise, m^6^A modifications and the role of regulators in OC are constantly being reported. At present, METTL3, YTHDF1, ALKBH5, etc., function as oncogenes in OC.^288–292^ METTL3 is frequently upregulated in OC. METTL3 expression is significantly correlated with clinicopathological features, such as tumor size, tumor grade, lymph node metastasis, pT status, pN/pM status, and FIGO stage.^288,289^ Liu et al.^290^ showed that YTHDF1 promoted OC progression by enhancing EIF3C translation. EIF3C is a subunit of the protein translation initiation factor EIF3. The proposal of the novel YTHDF1-EIF3C axis helps find effective targets and mechanisms for the treatment of OC.

#### Cervical and endometrial cancers

Cervical cancer (CC) is one of the most common gynecological malignancies, especially in developing countries.^293,294^ More than 90% of cervical cancers are squamous cell carcinomas.^295^ The early symptoms of cervical cancer are not obvious, and most patients are already in the late stage when they are found.^296^ Therefore, finding early cancer biomarkers is of great significance. As a way of posttranscriptional modification of RNA, m^6^A has stimulated the interest of researchers in recent years. Although there are few reports on m^6^A and CC, it is undeniable that m^6^A modification and its regulators play an important role in affecting CC progression.^297–299^ FTO is upregulated in CC, which not only affects the biological process of CC but also plays a role in chemotherapy resistance.^298,299^ In endometrial cancer, studies also found the role of m^6^A modification. m^6^A mRNA methylation promotes the proliferation of endometrial cancer by regulating Akt activity and plays a role in promoting cancer.^300^

### m^6^A and nervous system neoplasms

#### Glioblastoma (GBM)

GBM is the most lethal primary malignant brain tumor, commonly found in adults.^301^ GBM is characterized by significant intratumoural and intertumoral heterogeneity. GBM contains many GBM stem cell-like cells (GSCs) that can self-renew and have greater resistance to conventional treatments, radiotherapy, and chemotherapy.^301–303^ Therefore, it is necessary to further understand the molecular mechanism of GSC and find possible therapeutic targets. An increasing number of studies revealed the role of m^6^A RNA methylation in regulating GSC self-renewal and GBM occurrence.^304–306^ For example, ALKBH5 demethylates FOXM1 nascent transcripts, resulting in enhanced FOXM1 expression, thereby maintaining GSC tumorigenicity. In addition, METTL3 maintains carcinogenesis in GBM by mediating nonsense-mediated degradation of SRSF mRNA.^307^ In another study, 2HG inhibited tumor proliferation by targeting the FTO/m^6^A/MYC/CEBPA signaling pathway, demonstrating an antitumor effect.^308^

### m^6^A and hematological neoplasms

#### Acute myeloid leukemia (AML)

Normally, pluripotent hematopoietic stem cells differentiate into myeloid progenitor cells and eventually into mature myeloid cells, a process called myelopoiesis. Abnormal bone marrow production can lead to diseases, such as AML.^309,310^ AML, one of the most common and lethal hematopoietic malignancies, blocks its myeloid differentiation to generate self-renewing leukemia stem cells (LSCs).^311,312^ Abnormal cell proliferation and arrest of terminal differentiation of myeloid cells are the two hallmarks of AML.^313,314^ The development of specific targeted therapies for AML is a current priority. Emerging evidence suggested that RNA m^6^A modification is involved in various physiological and pathological processes, including hematopoiesis and leukemogenesis. At present, there are a lot of studies on m^6^A and AML, and m^6^A “writers,” “erasers,” and “readers” are involved in AML progression and play a role in promoting cancer.^315–319^ Genetic alterations in m^6^A regulators predicted poor survival in AML patients.^320^ m^6^A modifications and regulators mainly play important roles in the development and progression of AML and self-renewal of LSCs/leukemia-initiating cells. In terms of mechanism, METTL14 mainly regulates the expression of its mRNA targets MYB and MYC through m^6^A modification, thereby exerting its oncogenic effect. Furthermore, it is negatively regulated by SPI1. A novel SPI1-METTL14-MYB/MYC signaling axis plays an important role in myelopoiesis and leukemogenesis.^321^ In addition, Su et al.^308^ investigated the functional role of R-2-hydroxyglutaric acid (R-2HG), an oncogenic metabolite, in AML. Results revealed that R-2HG exerts broad antileukemic activity in vitro and in vivo by inhibiting AML cell proliferation and promoting cycle arrest and apoptosis. Mechanistically, R-2HG inhibits FTO activity and reduces the stability of MYC/CEBPA transcripts in an m^6^A-modified manner, resulting in the inhibition of related pathways. In conclusion, it is not difficult to see that m^6^A modification and its regulators mainly promote cancer in AML. Further search for molecules and sites targeting m^6^A to specifically manufacture related inhibitors may be expected to provide new therapies for AML diseases.

### m^6^A and tumors of other systems

In addition to its involvement in the aforementioned systems and cancers, m^6^A is widely involved in breast cancer,^322–324^ melanoma,^325–328^ osteosarcoma,^329,330^ head-and-neck SCC (HNSCC),^331–333^ etc. (summarized in Table 1). In melanoma, Jia et al.^328^ showed that m^6^A levels were low in ocular melanoma samples and predicted poor prognosis. Mechanistically, YTHDF1 promoted the translation of methylated HINT2 mRNA, which inhibited ocular melanoma progression. Another study revealed that METTL3-mediated m^6^A RNA methylation promotes uveal melanoma cell proliferation, migration, and invasion by targeting c-Met. m^6^A modification acts as a key oncogenic regulator in uveal melanoma development.^325^ The reason for the contradiction is unclear, but more research is needed to further verify and explain this phenomenon. Based on this, the regulatory mechanism of m^6^A modification is very complex. First, because there are many m^6^A regulators, each has different functions. In addition, there are many targets for m^6^A modification, and the biological effects of different targets are also very different. Second, m^6^A modifications are located on different regulatory mechanisms in different cells. Therefore, key enzymes of m^6^A modification may have opposite effects on tumorigenesis in different systems, even within one specific type of tumor.

## m^5^C writers, erasers, and readers

m5C refers to a methyl group inserted into the C atom at the fifth position of cytidine. In 1950, studies found m^5^C in nucleic acids. At that time, m^5^C was only found in the deoxypentose nucleic acids of animals and higher plants.^334^ In the 1970s, some research reports on m^5^C in RNA began to appear.^80,335,336^ So, far, m^5^C has become a research hotspot in RNA methylation modification. The technologies used to detect m^5^C are also constantly updated, currently mainly including bisulfite sequencing, m^5^C RNA immunoprecipitation sequencing (m^5^C-RIP-seq), 5-azacytidine-mediated RNA immunoprecipitation sequencing (Aza-IP-seq), and methyl CL-single nucleotide resolved cross-link immunoprecipitation sequencing (miCLIP-seq). Similar to m^6^A, m^5^C is also modified by three major regulators. “writers” refers to members of the DNA methyltransferase 2 (DNMT2) and NOP2/SUN RNA methyltransferase family member (NSUN) family. The latter mainly includes NSUN1, NSUN2, NSUN3, NSUN4, NSUN5, NSUN6, and NSUN7. The TET family and ALKBH1 constitute the “erasers” of m^5^C. As for “readers,” two main ones have been found, namely ALYREF and Y-box binding protein 1 (YBX1).

NSUN2 is the first discovered and the most thoroughly studied member of the NSUN family. For the first time, tRNA-specific methyltransferase 4 (Trm4) in yeast was involved in tRNA methylation modification.^337,338^ Since then, studies have identified the homologous protein of Trm4 in animals, now known as NSUN2.^339,340^ The process of NSUN2 catalyzing m^5^C methylation mainly involves the covalent binding of sulfur atoms of cysteine residues to the C6 position of the base in the target RNA, and then C5 nucleophilically attacks the methyl group of the SAM to complete the methylation.^341–344^ NSUN2-catalyzed methylation has been found in tRNA, mRNA, rRNA, mitochondrial tRNA (mt-tRNA), vault-derived small RNA, lncRNA, viral RNA, and other RNAs.^345–350^ In addition, conserved residues of the *NSUN2* gene undergo missense alterations in autosomal recessive mental retardation, revealing the role of RNA methyltransferases in human neurocognitive development.^351–353^ NSUN1 (or p120, also known as Nop2 in yeast) and NSUN5 (also known as Rcm1 in yeast) are mainly related to 28S rRNA in humans.^354–356^ NSUN4 is also mainly associated with rRNA. NSUN4 is a bifunctional mitochondrial protein required for 12S rRNA methylation and the filament assembly site.^357^ NSUN3 initiates 5-formylcytidine biogenesis in human mt-tRNA (Met).^358–360^ Another tRNA methylation regulator is NSUN6, which mediates the specific methylation of tRNA (Cys) and tRNA (Thr) located at position C72.^361^ The specific role of NSUN7 is not yet fully understood. However, the continuous development of next-generation sequencing (NGS) technology will lead to a deeper understanding of the regulators of m^5^C in the future. DNMT2 (also known as TRMDT1) is the most widely studied methyltransferase besides NSUN2. DNMT2 does not only act on tRNA. tRNA (Asp-GTC), tRNA (Val-AAC), and tRNA (Gly-GCC) are all its substrates. It also catalyzes mRNA methylation.^345,362–365^

**Fig. 4:**
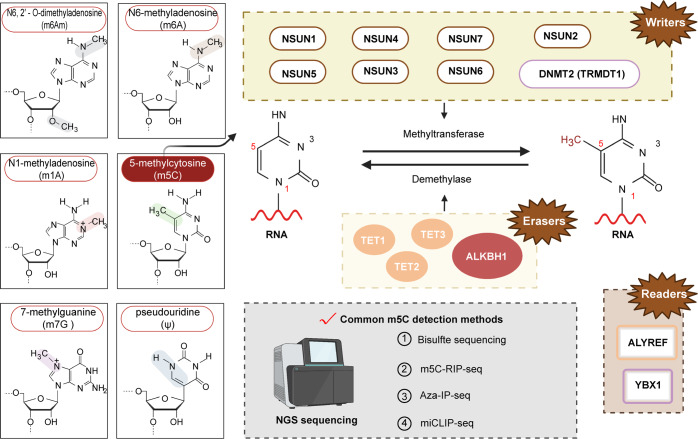
The mechanism of m^5^C regulation and the common detection methods. Image created with BioRender (https://biorender.com/)

Compared to the writers of m^5^C, research on erasers and readers is relatively less mature. The 10–11 translocation (Tet) family of mammalian Fe(II)- and 2-oxoglutarate-dependent dioxygenases can oxidize RNA 5-methylcytidine to 5-hydroxymethylcytidine (5hmrC), 5-formylcytidine (5frC), and 5-carboxycytidine (5carC).^366^ Tet2 promotes myelopoiesis induced by mammalian pathogen infection by reducing m^5^C in mRNA.^367^ In addition, studies revealed a full-length isoform containing an N-terminal CXXC domain (Tet3FL). The CXXC domain binds unmethylated CpGs, but it has the highest affinity for 5caC but not m^5^C.^368^ Another currently known eraser is ALKBH1. m^5^C modification can be further oxidized by α-ketoglutarate and the Fe(II)-dependent dioxosome ALKBH1/ABH1 to generate 5-formylcytidine at this position.^360^ ALYREF is a reader protein that specifically recognizes m^5^C, which binds mainly to the 5′ and 3′ regions of mRNA in vivo.^369^ m^5^C RNA modification promotes retroviral replication in an ALYREF reader protein-dependent manner.^370^ In addition, studies revealed that, upon NSUN2 depletion, ALYREF-mediated dysregulation of mRNA output could be restored by reconstitution of wild-type but not methyltransferase-deficient NSUN2.^346^ In addition to ALYREF, YBX1, a newly discovered m^5^C reader protein, regulates mRNA stability in the cytoplasm (Fig. 4).^371^

## Functional consequences of m^5^C in coding RNAs and ncRNAs

Initial studies of m^5^C in RNA were mainly limited to tRNA and rRNA. RNA bisulfite conversion was combined with NGS technology to whole-transcriptome detection of modified cytosine residues at single-nucleotide resolution, which found 10,275 m^5^C sites in mRNA and other ncRNAs in addition to tRNA and rRNA. This study provides the first map of the distribution of m^5^C modifications in the human transcriptome, advancing the study of posttranscriptional modifications in gene regulation.^372^ Next, this review mainly summarized the action sites and effects of m^5^C on tRNA, rRNA, mRNA, and other ncRNAs and briefly described the mechanism, hoping to provide some help for further research in the future.

### Role of m^5^C in tRNA

tRNA acts as a carrier for amino acids, providing activated amino acids during protein synthesis.^373^ tRNA is generally composed of 74 to 95 nucleotides and has a stable spatial structure.^374,375^ The methylation sites of tRNA are mainly located at C34, C38, C40, C48, and C50 of the anticodon loop.^376–378^ m^5^C promotes the stability of tRNA for subsequent protein synthesis. Specifically, m^5^C methylation from tRNA in mice was eliminated by disrupting DNMT2 and NSUN2 tRNA methyltransferases. A dramatic reduction in the steady-state levels of unmethylated tRNA and a reduction in the overall protein synthesis rate was observed.^345^ Gkatza et al.^379^ found that loss of NSUN2 resulted in reduced methylation of tRNA-derived noncoding fragments, further resulting in impaired regulation of protein synthesis. Furthermore, NSUN2-driven RNA methylation is functionally required to adapt cell cycle progression to early stress responses. Thus, cytosine-5 RNA methylation links protein synthesis to cellular metabolism. m^5^C also affects tRNA cleavage, resulting in neurodevelopmental disorders.^380^ tRNAs with DNMT2-dependent methylation can discriminate homologous codons to accurately direct polypeptide synthesis and protect themselves from stress-induced endonucleolytic cleavage.^363,364^

m^5^C also plays an important role in mt-tRNA. NSUN3 interacts with (mt-)tRNA^Met^ and undergoes methylation modification at its C34 wobble position, expanding codon recognition during mitochondrial translation. In addition, NSUN3 specifically recognizes the anticodon stem-loop (ASL) of tRNA, and impairing the ASL base pairing causes disease.^360^ NSUN3 mutation also causes insufficient formylation at the same site of mt-tRNA.^358^ NSUN2 also targets mt-tRNA positions 48–50 to catalyze the formation of m^5^C methylation. However, studies revealed that NSUN2 does not affect mt-tRNA stability and oxidative phosphorylation.^347,381^

### Role of m^5^C in rRNA

rRNA and ribosomal proteins make up the ribosome, which provides a site for protein synthesis in vivo.^382^ rRNA accounted for about 80% of the total RNA weight.^383,384^ rRNA methylation plays an important role in mitochondrial ribosome biogenesis. Studies first found that yeast contains two m^5^C modification sites, 2278 of helix 70 and 2870 of helix 89, respectively. Further, Rcm1 and Nop2 were responsible for catalyzing the methylation of these two sites, and the cysteines in motifs IV and VI of these two proteins played an important role.^355^ Gigova et al.^385^ also revealed that methylation of the 25S rRNA IV domain is critical for ribosome stability. In addition, in humans, NSUN1 and NSUN5 catalyze m^5^C4447 and m^5^C3782 of 28S rRNA, respectively.^355^ NSUN5 also helps generate specialized ribosomes that fight stress, extending lifespan and stress resistance in yeast, worms, and flies.^386^ First, NSUN4 methylates the 12S rRNA of the small ribosomal subunit, and sequencing results showed that the methylation site is at cytosine 911. More importantly, NSUN4 can also cooperate with MTERF4 to promote ribosome assembly. Thus, NSUN4 plays a key role in controlling the final step of ribosome biogenesis.^357,387^ More interestingly, the YTHDF2 protein is also one of the readers of m^5^C in RNA, although its affinity is not as strong as that of m^6^A. RNA bisulfite sequencing results showed that YTHDF2 deletion significantly enhanced m^5^C methylation levels in rRNA. It is also involved in pre-rRNA processing.^388^ In conclusion, m^5^C modification in rRNA plays a role in ribosome assembly, synthesis, and stability, regulates cellular responses to stress, and prolongs lifespan.

### Role of m^5^C in mRNA

In early zebrafish embryos, genome-wide analysis of RNA m^5^C modifications revealed that m^5^C-modified maternal mRNAs exhibit higher stability than m^5^C-unmodified mRNAs. The mechanism is as follows: YBX1 acts as an m^5^C reader, preferentially recognizes m^5^C-modified mRNAs through the indole loop of the W65 cold shock domain, and recruits Pabpc1a or ELAVL1 (both mRNA stabilizers) to maintain the stability of its target mRNAs.^371,389^ m^5^C also promotes mRNA export, and ALYREF is another m^5^C reader. Yang et al.^346^ revealed the role of ALYREF and NSUN2 in regulating mRNA output. In addition, the combined modification of m^5^C and m^6^A on the same RNA will produce a synergistic effect and jointly affect the expression of subsequent proteins. Specifically, NSUN2-mediated m^5^C and METTL3/METTL14-mediated m^6^A methylation synergistically enhance p21 mRNA expression.^390^ Schumann et al.^391^ showed that cytosine methylation levels are strongly negatively correlated with mRNA translation. The contradiction between the two was explained by different m^5^C profiles. Huang et al.^392^ also pointed out that species, rather than tissue type, is the main determinant of methylation levels. In addition, downregulation of TRDMT1, a tRNA methyltransferase, affected mRNA methylation levels, further inhibiting cancer proliferation and migration.^393^ In conclusion, m^5^C mainly affects mRNA stability, export, translation level, and subsequent biological functions, and the critical role of m^5^C mRNA modification in early development.

### Role of m^5^C in other ncRNAs

Cytosine methylation can also be involved in the regulation of lncRNA function. lncRNA HOTAIR and XIST are specific targets of m^5^C. The m^5^C level in the XIST A structure significantly affects the binding of XIST to the chromatin modification complex PRC2.^394^ In addition, NSUN2 mediates m^5^C-modified lncRNA nuclear magnetic resonance (NMR) by competitively inhibiting the methylation of underlying mRNAs. lncRNA NMR plays an oncogenic role in ESCC.^349^ In addition, m^5^C was also deposited in the noncoding vault RNA VTRNA1.1. NSUN2 mediates the methylation of cytosine 69 of VTRNA1.1, thereby processing VTRNA1.1 into small vault RNAs (svRNAs). svRNAs can regulate epidermal differentiation and play an important role in cell differentiation.^348^ Likewise, NSUN2 is the primary writer of m^5^C on HIV-1 RNA. NSUN2 deletion not only inhibits m^5^C modification of HIV-1 transcripts but also inhibits HIV viral replication.^395^ DNMT2 deletion in mice prevents m^5^C modification in sperm miRNAs and abrogates small ncRNA-mediated intergenerational transmission of metabolic disorders.^396^

## Role of m^5^C in cancer

Recently, there has been a growing body of research on m^5^C and cancer, although the specific mechanisms of m^5^C in some cancers remain obscure. Current and future research will increasingly clearly describe the role and molecular mechanism of m^5^C in cancer. Misu is the first SUN domain-containing protein discovered in invertebrates, and its expression level in normal tissues is much lower than in tumor tissues. Misu can affect the role of Myc, a well-known proto-oncogene, in tumor cell growth and proliferation.^338^ In addition, NSUN2, with high sequence homology to Misu, was overexpressed in almost all cancers.^397^ Xiang et al.^398^ conducted the first comprehensive analysis of m^5^C regulators in gastrointestinal cancer. Also, m^5^C regulators were closely related to the ErbB/PI3K-Akt signaling pathway. This review summarized the current research on m^5^C and cancer and discussed the basic roles and possible molecular mechanisms of m^5^C by classifying the digestive and nondigestive system cancers, hoping to provide some help for future research (Table 2).

**Table 2:** RNA modifying regulators of m^5^C in human cancers

	Cancer type	Category	m^5^C regulators	Expression (tumor vs. normal)	Property	Prognostic implication of m^5^C regulators overexpression	Functional role	Molecular mechanism	Related genes	Refs.
Digestive system	Hepatocellular carcinoma	Writer	NSUN4	Upregulation	Oncogene	Poor	Associated with methylation and demethylation processes	/	/	402
		Reader	ALYREF	Upregulation	Oncogene	Poor	Associated with cell cycle regulation and mitosis	/	/	402
		Writer	NSUN2	Upregulation	Oncogene	/	Associated with poor differentiation	Mediates H19 m^5^C modification	LncRNA H19, G3BP1, MYC	403
	Gastric cancer	Writer	NSUN2	Upregulation	Oncogene	/	Promotes cell proliferation in vitro and in vivo	Methylates and inhibits the expression of p57^Kip2^	p57^Kip2^	404
		Writer	NSUN2	Upregulation	Oncogene	Poor	Promotes cell proliferation, migration, and invasion in vitro	SUMO-2/3 stabilizes NSUN2 and mediates its nuclear transport	SUMO-2/3, PIK3R1, and PCYT1A	72
	Pancreatic cancer	Writer	NSUN6	Downregulation	Tumor suppressor	Favorable	Associated with Ki67^+^ cell rate, inhibits cell proliferation in vitro and in vivo	/	/	409
	Esophageal squamous cell carcinoma	Writer	NSUN2	/	Oncogene	/	Associated with cancer progression	Mediates NMR m^5^C modification	LncRNA NMR	349
		Writer	NSUN2	Upregulation	Oncogene	Poor	Promotes cell proliferation, migration, and invasion	Stimulates oncogenic PI3K/AKT and ERK/MAPK signaling by enhancing GRB2 mRNA stability	E2F1, GRB2	411
	Colon carcinoma	Writer	NSUN6	Upregulation	Oncogene	Poor	Associated with TIME	/	/	412
		Writer	DNMT2	Upregulation	Oncogene	Poor	Associated with TIME	/	/	412
		Eraser	ALKBH1	Upregulation	Oncogene	Poor	Associated with TIME	/	/	412
	Gallbladder carcinoma	Writer	NSUN2	Upregulation	Oncogene	/	Associated with advanced clinical stage, promotes cell proliferation in vitro and in vivo	Synergizes with RPL6	RPL6	71
Non-digestive system cancer	Glioma	Writer	NSUN5	Downregulation	Tumor suppressor		Epigenetic loss of NSUN5 inhibits protein synthesis while activating stress-adapted translational programs	/	/	416
	Lung adenocarcinoma	Writer	NSUN1	Upregulation	Oncogene	Poor	/	/	/	417
	Lung squamous cell carcinoma	Writer	NSUN3	Upregulation	Oncogene	Poor	Associated with immune infiltration	/	/	418
		Writer	NSUN4	/	Oncogene	Poor	Associated with immune infiltration	/	/	418
	Breast cancer	Writer	NSUN2	Upregulation	Oncogene	/	Associated with clinical stage, tumor classification, pathological differentiation, the expression levels of ER, PR, and Ki-67, promotes cell proliferation, migration, and invasion	/	/	419
		Reader	YBX1	/	/	/	Regulates estrogen response	Interacts with ER receptor and inhibits its activity	NFIB, FGFR2	421
	Bladder cancer	Reader	YBX1	Upregulation	Oncogene	Poor	Associated with advanced T and N stages and tumor grade	Recruits ELAVL1 to maintain the stability of m^5^C-modified RNAs	ELAVL1, HDGF	371
		Writer	NSUN2	Upregulation	Oncogene	Poor	Associated with advanced T and N stages and tumor grade	NSUN2 and YBX1 exert oncogenic roles by targeting m^5^C methylation in the 3’ untranslated region of HDGF	HDGF	371
	Leukemia	Writer	NSUN1	/	Oncogene	/	Mediates the generation of 5-AZA-insensitive chromatin structures	Synergizes with BRD4 and RNA-polymerase-II	BRD4	429
		Writer	NSUN3	/	Tumor suppressor	/	Mediates the generation of 5-AZA-sensitive chromatin structures	Binds hnRNPK and recruites RNA polymerase-II at nascent RNA with CDK9/P-TEFb	hnRNPK, CDK9/P-TEFb	429
		Writer	DNMT2	/	Tumor suppressor	/	Mediates the generation of 5-AZA-sensitive chromatin structures	Binds hnRNPK and recruites RNA polymerase-II at nascent RNA with CDK9/P-TEFb	hnRNPK, CDK9/P-TEFb	429
	Prostate carcinoma	Writer	NSUN1	Upregulation	Oncogene	Poor	/	/	/	430
	Head and Neck Squamous Carcinoma	Writer	NSUN2	Upregulation	Oncogene	Poor	/	/	/	431
	Ovarian cancer	Writer	NSUN2	Upregulation	/	Not necessarily	Synergistically with IGF-II to affect the survival and prognosis of ovarian cancer patients	The NSUN2^low^IGF-II^high^ subgroup has the worst survival	IGF-II	432

### m^5^C and digestive system cancers

5 mC profiling of mRNA, circRNA, and lncRNA in human HCC and adjacent normal tissues was carried out.^399–401^ No matter which kind of RNA m^5^C was modified, there was a significant difference between cancer and paracancer. Despite the lack of follow-up molecular investigations, these expression profiles are important for understanding the mechanisms of m^5^C regulation in HCC. Through bioinformatics analysis, He et al.^402^ revealed the characterization and role of m^5^C regulators in HCC and pointed out that m^5^C-related gene mutations are prevalent in HCC. In addition, more m^5^C regulators are associated with the clinical grade and prognosis of HCC. Among them, NSUN4 and ALYREF were effective predictors of survival. Another study found that NSUN2 mediated the m^5^C modification of H19, promoted the specific binding of H19 to G3BP1, and further led to MYC accumulation, which subsequently promoted the occurrence, development, and poor differentiation of HCC.^403^

NSUN2 expression was increased in GC tissues. NSUN2 could improve GC cell proliferation in vivo and in vitro through a series of experiments, such as CCK-8 assay, colony formation assay, flow cytometry analysis, and nude mice tumorigenesis experiment. Mechanistically, NSUN2 methylated the 3′-UTR of CDKN1C (p57^Kip2^) mRNA, which led to p57^Kip2^ downregulation.^404^ Another study on NSUN2 and GC revealed that high NSUN2 expression was closely related to poor prognosis in GC patients. Similarly, NSUN2 also functions as an oncogene in GC cells. SUMO-2/3 interacted with NSUN2 to maintain the stability of NSUN2 and promoted its nuclear translocation, thereby enhancing the cancer-promoting effect.^72^ Although only the role of m^5^C writer in GC is currently studied, with technological progress and research accumulation, the role of m^5^C regulators in GC will be clearer in the future.

PC is the most dangerous type of cancer. Mortality is very high, and the prognosis is very poor.^405–407^ The role of RNA posttranscriptional modifications in PC is constantly being updated and presented. The expression profile of m^5^C regulators in PC has been reported.^408^ The differential expression of m^5^C-related genes in PC suggested that different regulators play different or even opposite roles. NSUN6 is a tumor suppressor gene in PC, and its expression was closely correlated with clinicopathological features, such as T stage and Ki-67^+^ cell rate. Moreover, NSUN6 can also predict the prognosis and recurrence of PC, and it is expected to become an excellent target for the prognosis evaluation of cancer diagnosis and treatment.^409^ Yuan et al.^410^ also constructed a prognostic risk model for 8-m^5^C-related lncRNAs in pancreatic ductal adenocarcinoma. Although this prognostic model is the first to be constructed, it does have a certain reference value. However, the disadvantage is that this model has not been validated by in vivo experiments. Therefore, in-depth mechanistic studies and validation in the future will better reveal the role of m^5^C regulators in PC.

Two studies reported the role of m^5^C-related regulators in ESCC. Both revealed the cancer-promoting role of m^5^C writer NSUN2 in ESCC. The first study showed that NSUN2 was involved in how lncRNA NMR promotes tumor progression in ESCC. NSUN2 methylation modifies NMR, which binds to the chromatin regulator BPTF to initiate downstream pathways.^349^ The other study found that NSUN2 exerted a procancer effect by stimulating oncogenic PI3K-Akt and extracellular signal-regulated kinase (ERK)/mitogen-activated protein kinase (MAPK) signaling by enhancing GRB2 mRNA stability.^411^

Similar bioinformatics studies were also conducted on colon cancer. Based on RNA sequencing data of The Cancer Genome Atlas dataset, a prediction model containing three m^5^C regulators, NSUN6, DNMT2, and ALKBH1, was established. The expression of these three regulators is highly correlated with colon cancer prognosis. In addition, NSUN6, DNMT2, and ALKBH1 can work together on the MAPK signaling pathway, thereby affecting the immune infiltration level in colon cancer.^412^

NSUN2 is one of the most well-studied and extensively studied m^5^C regulators. As summarized above and demonstrated in this study, NSUN2 is closely related to cell immortalization.^338^ Therefore, Gao et al.^71^ explored the role of NSUN2 in gallbladder carcinoma (GBC). The result was that NSUN2 cooperated with RPL6 to promote GBC cell proliferation and growth. In conclusion, m^5^C is involved in various processes in digestive system cancer development. Current studies supported the cancer-promoting role of m^5^C in HCC, GC, ESCC, colon carcinoma, and GBC. The protective effect of the tumor suppressor was demonstrated only in PC. In addition, among m^5^C-related regulatory genes, the writer is currently the most studied and reported, and NUSN2 among writers is the most extensively studied and the most clearly described in function. More and more relevant studies in the future will further reveal the role and possible molecular mechanisms of m^5^C in digestive system cancers, providing as many clues as possible for cancer diagnosis, treatment, and prognosis evaluation.

### m^5^C and nondigestive system cancers

#### Glioma

The construction of the prognostic prediction model of RNA m^5^C methyltransferase in glioma has also been completed. Data from existing databases were obtained, processed, and evaluated using bioinformatics. There is a certain link between the abnormal expression of m^5^C methyltransferase and the clinicopathological features of glioma. Except for NSUN6, the expression of other methyltransferases was upregulated with the increase of the WHO grade. Expression levels were also positively correlated with the malignant progression of glioma.^413^ This study deepens the understanding of the molecular mechanisms by which m^5^C is involved in the occurrence and development of gliomas and provides possible ideas for finding new biomarkers and targeted therapies. GBM is the most malignant type of glioma.^414^ Cheray et al.^415^ investigated the biological role and underlying mechanism of m^5^C modification of miRNAs in GBM. Specifically, DNMT3A/AGO4 inhibits miR-181a-5p/mRNA duplex formation by mediating cytosine methylation of miR-181a-5p. The original function of miRNA to inhibit mRNA gene expression is lost. The methylation status of miR-181a-5p also affects its interaction with antiapoptotic proteins, resulting in enhanced cancer cell proliferation and invasion and reduced apoptosis. Furthermore, cytosine methylation of miR-181a-5p was also associated with poor prognosis in GBM patients. Epigenetic deletion of NSUN5 affects protein synthesis, on the one hand, and targets the ribosome to activate alternative translation programs involved in stress-adaptive responses, on the other. Epigenetic inactivation of NSUN5 is a marker of long-term survival in glioma patients.^416^

#### Lung cancer

Nucleolar protein p120 is also known as NSUN1. High p120 expression predicted poor clinical outcomes compared to LUAD patients with low p120 expression. Multivariate analysis showed that p120 is the independent and strongest prognostic factor for resected LUAD (*P* = 0.033).^417^ Bioinformatic analysis methods were also applied to explore the characterization and impact of m^5^C modification on RNA in LUSC. Most m^5^C regulators were upregulated in LUSC, with only DNMT2 and NSUN7 having decreased expression levels compared to normal tissues. Among them, NSUN3 and NSUN4 were associated with clinicopathological features and survival of LUSC. In addition, they were also closely associated with the p53, cell cycle, and mTOR signaling pathway. In exploring the relationship between m^5^C regulators in LUSC and the tumor immune microenvironment, NSUN3 was mainly closely linked to CD8^+^ T cells, whereas NSUN4 was closely linked to neutrophils. In conclusion, m^5^C regulators have a major role in predicting the prognosis of LUSC patients and regulating the immune microenvironment.^418^ In addition, Pan and Chen also constructed prognostic prediction models of m^5^C modification in LUAD patients, respectively.^48,66^

#### Breast cancer (BRC)

Some studies reported the role of m^5^C modification-related lncRNAs in BRC. Risk models based on three m^5^C-lncRNAs (AP005131.2, AL121832.2, and LINC01152) can be used to predict the survival and prognosis of BRC patients. These three lncRNAs are expected to be novel markers and therapeutic targets for BRC. Yi et al.^419^ revealed that NSUN2 is involved in the metastatic progression of BRC. NSUN2 content in BRC was higher than in adjacent normal tissues. NSUN2 expression was closely related to many pathological features, including estrogen receptor (ER) and progesterone receptor. In addition, as in other cancers, NSUN2 also played a cancer-promoting effect in BRC, promoting BRC cell proliferation in vitro and in vivo and cell migration and invasion in vitro. Proliferation-associated nucleolar antigen p120, also as an RNA methyltransferase, also affects BRC prognosis, but the specific mechanism remains to be further explored.^420^ In addition to the m^5^C writer, the reader protein plays a role in BRC. Campbell et al.^421^ found that YBX1 interacted with the ER receptor and inhibited its activity, altering the estrogen dependence of BRC cells. FGFR2 signaling enhances this interaction, collectively transforming BRC into an estrogen-negative form. In conclusion, m^5^C-related regulators are expected to be new targets for BRC therapy.

#### Others

Models of m^5^C-related regulators for predicting prognosis in cancers, including PCa, HNSCC, OC, oral squamous cell carcinoma (OSCC), papillary thyroid carcinoma, and ccRCC, are continuously being established.^422–428^ In addition, some studies are based on the establishment of models and in-depth in vitro and in vivo experiments to further verify the role of m^5^C. For example, m^5^C promoted BLC progression. Specifically, the m^5^C reader YBX1 promoted the stability of its targeted mRNAs by recruiting ELAVL1. NSUN2 and YBX1 exerted oncogenic roles by targeting m^5^C in the 3′-UTR of HDGF. The proposal of the NSUN2/YBX1/m^5^C-HDGF signaling axis, to a certain extent, revealed the potential molecular mechanism in promoting the development of BLC.^371^ In leukemia, RNA m^5^C modifications and methyltransferases affected the chromatin structure, affecting patient response to drugs. NSUN3 and DNMT2 bind hnRNPK, which interacts with the transcription factor GATA1, and SPI1/PU.1, and with CDK9/P-TEFb to recruit RNA polymerase II at nascent RNAs to generate the 5-AZA-sensitive chromatin structure. In contrast, NSUN1 forms an active, 5-AZA-insensitive chromatin structure mainly with BRD4 and RNA polymerase II.^429^ High p120 (NSUN1) expression in PCa was related to increased tumor aggressiveness and poor prognosis.^430^ Similarly, NSUN2 was upregulated in HNSCC, and high NSUN2 expression was closely associated with poor clinical outcomes. NSUN2 can be used as a potential prognostic marker for HNSCC, providing a reference for finding new treatments.^431^ Interestingly, in OC, studies reported for the first time that NSUN2 and insulin-like growth factor-II (IGF-II) synergistically affected patient survival and prognosis. The NSUN2^low^IGF-II^high^ subgroup had the worst survival, whereas the NSUN2^high^IGF-II^low^ subgroup had the best overall and progression-free survival.^432^

## Ψ and Ψ synthase

Ψ is the C5-glycoside isomer of uridine. The normal pyrimidine nucleoside is the N-1 atom of the heterocyclic ring bonded to the C-1′ atom of the pentose to form a glycosidic bond, whereas the pseudouracil nucleoside is the C-5 atom of the heterocyclic ring bonded to the C-1′ atom of the pentose.^433^ Ψ is the first discovered and currently the most abundant modified nucleoside in RNA, known as the “fifth nucleoside” in RNA.^434–436^ As early as 1951, Cohn et al.^437^ first obtained the complete 5′ nucleotide by enzymatic hydrolysis of calf liver ribonucleic acid. It was later named Ψ (psi, Ψ).^438,439^ Initially, Ψ was mainly reported in ncRNAs, such as rRNAs, tRNAs, and small nuclear RNAs (snRNAs). With the update of detection methods and technologies, Ψ modified almost all RNAs, including mRNA.^440,441^ The current commonly used detection methods for Ψ mainly include traditional reverse transcriptase and gel electrophoresis methods and methods based on mass spectrometry (MS; matrix-assisted laser desorption/ionization-MS combined with chemical derivatization).^442–444^

The conversion of uridine to Ψ is catalyzed by Ψ synthase, also called pseudouridine synthase (PUSs) in eukaryotes, the writer of Ψ. So far, six Ψ synthase families have been identified, namely TruA, TruB, TruD, RsuA, RluA, and Pus10p. Although these enzymes differ greatly in sequence, they all share a conserved core structure and active site.^445–447^ In yeast, the TruA family mainly includes Pus1, Pus2, and Pus3. Pus4 is the only member of the TruB family. The RluA family of enzymes includes Pus5, Pus6, Pus8, and Pus9. There is only one member of the TruD family, Pus7. In addition, the human homologs of PUSs are PUS1-4, PUS6, PUS7, PUS7L, PUS9, PUS10, RPUSD1-4.^75,448,449^ Another writer is dyskerin (DCK1), a H/ACA small ribonucleoprotein guide RNA-dependent enzyme that catalyzes the formation of Ψ using a guide RNA and protein (Cbf5 in yeast and DKC1 in humans) complementary to the sequence to be modified.^436,450,451^

As mentioned above, there are two main ways of pseudouracilylation of RNA substrates in eukaryotes. One is an RNA-independent mechanism whereby PUSs directly recognize and catalyze substrates. The other is an RNA-dependent mechanism that requires catalysis by box H/ACA RNPs.^452^ Each RNP contains a unique guide RNA component and a set of evolutionarily conserved four core proteins. The latter mainly includes dyskerin (DKC1; NAP57 in rats and Nop60B in *Drosophila*), nonhistone 2, nucleolar protein 10, and glycine-arginine-rich protein 1. The box H/ACA small RNPs complex recognizes the substrate and plays a role in the base pairing of the substrate RNA, of which DKC1 has catalytic activity. The RNA-dependent mechanism of pseudouracilylation occurs mainly on ncRNAs.^453^

**Fig. 5:**
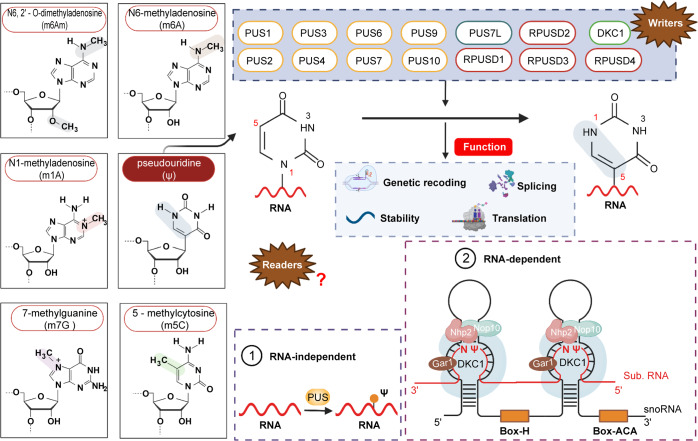
The mechanism of Ψ regulation. There are two main ways of pseudouracilylation of RNA substrates in eukaryotes. One is an RNA-independent mechanism whereby PUSs directly recognize and catalyze substrates. The other is an RNA-dependent mechanism that requires catalysis by box H/ACA RNPs. Each RNP contains a unique guide RNA component and a set of evolutionarily conserved four core proteins. Among them, only DCK1 has catalytic activity. Nhp2, Nop10, and Gar1 are regulatory units. Image created with BioRender (https://biorender.com/)

No specific “readers” and “erasers” for Ψ have been found. The absence of eraser protein is explained by the fact that the C–C bond formed between the base and the ribose sugar in Ψ is much more inert than the C–N bond, making the pseudouridylation process irreversible (Fig. 5).^454^

## Functional consequences of Ψ in coding RNA and ncRNAs

As mentioned above, Ψ was originally reported mainly in tRNA, rRNA, and snRNA. In 2014, Schwartz et al.^440^ and Carlile et al.^441^ performed whole-genome sequencing of Ψ. They detected numerous pseudouridylation sites on mRNA and ncRNAs. Schwartz et al. reported 328 unique Ψ sites in yeast mRNA and ncRNA. Carlile et al. revealed ~260 Ψ sites in 238 protein-coding transcripts in yeast and 96 Ψ sites in 89 mRNAs in humans. Furthermore, they revealed that Pus1, Pus2, Pus4, and Pus7 were involved in mRNA pseudouridylation. More importantly, they also found that this pseudouridylation modification was affected by factors, such as the environment, which affected the subsequent regulation of the targeted RNA. In the Ψ structure, the typical N-C bond between the ribose and the base is replaced by a C–C bond, resulting in the retention of the H at the N1 position, which is equivalent to creating an additional hydrogen bond donor. When incorporated into RNA, Ψ can affect RNA thermodynamic stability and spatial conformation by increasing base stacking, improving base pairing, and sclerosing the sugar-phosphate backbone, making RNA more stable.^454–459^ In addition to the above effects, Ψ is involved in tRNA codon-anticodon base pairing, rRNA folding, snRNP biogenesis, pre-mRNA splicing, mRNA encoding, and translation, stress response, translation fidelity, and peptide bond formation rate.^435,440,449,460,461^

### Role of Ψ in tRNA

In tRNA, there are a large number of pseudouridylation sites. Ψ can exist not only in the stem and loop of tRNA anticodon but also in TΨC loop and D stem, etc. For example, position 55 of the TΨC stem-loop structure, position 13 on the D stem, and positions 38 to 40 on the ASL structure.^462,463^ All these help stabilize the spatial structure of tRNA, coordinate codon and anticodon recognition pairings, and improve translation efficiency and accuracy.^447,449,464^ Huang et al.^465^ found that Asp60 mutations, conserved in nearly all Ψ synthases, affect catalytic activity. Furthermore, Asp residues are involved in the nucleophilic catalytic mechanism. However, the specific molecular mechanism is still being explored. A missense mutation in the *PUS1* gene affecting highly conserved amino acids affects Ψ synthase 1 (Pus1p) production and depletes the tRNA for pseudouridylation. This eventually leads to mitochondrial myopathy and sideroblastic anemia. A yeast strain with disrupted Pus3p (Deg1p) enzymatic activity fails to form Ψ at tRNA 38 and 39, whereas other sites are unaffected. Ψ in tRNA ALSs are important for regulating translation processes in yeast.^466^ In addition, Ψ can stabilize the ASL of tRNA^Lys,3^.^467^ The product of the yeast YNL292w (*PUS4*) gene was identified as an enzyme that catalyzes pseudouracil formation at position 55 in the tRNA molecule. As mentioned earlier, Pus4 is the only member of the TruB family, which is generally conserved among tRNAs.^468^ Another study demonstrated that the RNA Ψ synthase TruB catalyzes Ψ formation at U55 in tRNA. This modification mainly occurs in the T-arm of tRNA.^469^

### Role of Ψ in rRNA

Ψ is especially widely distributed in rRNA and present on almost all rRNAs. Ψ is distributed in different functional regions, mainly including the interface of large and small ribosomal subunits, the stem-loop structure of tRNA, the site of interaction with mRNA, the decoding center, and the peptidyl transferase center. Ψ affects ribosome production and protein synthesis. Box H/ACA small nucleolar ribonucleoproteins (snoRNAs) play a nonnegligible role in rRNA pseudouridylation.^461,470,471^ snoRNA can also regulate rRNA folding.^472,473^ In addition, Cbf5p (dyskerin) depletion also causes pre-rRNA processing defects similar to snR30 depletion.^474^ One study found that dyskerin deficiency in human cells results in defective rRNA uridine modification, altering ribosome activity.^475,476^ The gene encoding dyskerin is well-known as *DKC1*, and mutations in this gene can lead to Ψ deficiency and cause X-linked keratosis congenital.

### Role of Ψ in mRNA

Although the modification site of Ψ was found in mRNA much later than in other ncRNAs, many studies focused on the interaction and mechanism between Ψ and mRNA. Ψ can affect mRNA stability. In vitro transcribed mRNA contain in exhibited greater stability when transferred to in vivo studies than in vitro transcribed mRNA containing uridine.^477^ Another study also demonstrated that PUS7 deletion reduced pseudouracilated mRNA levels after heat shock in yeast, suggesting that it was beneficial for enhanced transcript stability.^440^ The artificial addition of Ψ can mediate nonsense codon transitions in mRNA and inhibit translation termination, which may be a new mechanism for causing protein diversity.^452^ Ψ also promotes pre-mRNA splicing. Specifically, snRNAs are involved in the maturation of eukaryotic mRNAs. They are mainly responsible for the splicing of pre-mRNA. Prp5 is an RNA-dependent ATPase involved in monitoring U2 BSRR-branch site base pairing interactions. Results suggested that Ψ in U2 snRNA promotes pre-mRNA splicing by directly altering Prp5 binding.^478–480^ In addition, Ψ also plays a very important role in mediating mRNA translation. When mice were injected with Ψ-encoded mRNA for erythropoietin, red blood cell production increased, indicating that in vitro transcribed mRNA containing the modified nucleoside Ψ is beneficial for enhanced translation.^481^ A follow-up study found that this translational enhancement was mediated by reduced PKR activation.^482^ Based on the above-mentioned role of Ψ in mRNA, many therapeutic approaches based on Ψ-mRNA have emerged.^477,481,483^ For example, Ψ incorporation into mRNA produces excellent nonimmunogenic vectors. It also enhances the translation ability and stability of mRNA.^477^

### Role of Ψ in other ncRNAs

Ψ is also present on lncRNA. For example, lncRNA ZFAS1, telomerase RNA component (TERC), and small nucleolar RNA host genes 1 and 7 (SNHG1 and SNHG7) contain pseudouracil components, but the specific mechanism remains to be confirmed by further studies.^461,484–486^ In conclusion, Ψ is the most abundant modification in RNA, and exploring the mechanism behind it is a challenge and an opportunity. Further studies will uncover more about the role and significance of Ψ in RNA.

## Role of Ψ in cancer

As mentioned above, Ψ is the most prevalent posttranscriptional modification in RNA. The pseudouridylation process is mainly catalyzed by PUSs, and there are two catalytic modes in eukaryotes. One is an RNA-independent way, which is acted independently by the PUSs. However, there are few reports about PUSs in cancer. The other is an RNA-dependent mechanism involving an enzyme called dyskerin encoded by the *DKC1* gene. It forms a complex with box H/ACA snRNA, pseudouridylates RNA, and mediates the posttranscriptional modification of RNA. In addition, dyskerin is also associated with human telomerase RNA containing the H/ACA RNA motif.^486–489^ DKC1 mutations cause dyskeratosis congenita (DC), a disease characterized by increased tumor susceptibility and a propensity to age.^487^ It was initially thought that lower pseudouridylation levels were a pervasive feature of cancer, in which DKC1 primarily functions as a tumor suppressor.^490^ However, more and more studies found that the DKC1 expression level is upregulated in some cancers and can also function as an oncogene (Table 3).^491,492^

**Table 3:** RNA modifying regulators of Ψ in human cancers

	Cancer type	Category	Ψ regulators	Expression (tumor vs. normal)	Property	Prognostic implication of Ψ regulators overexpression	Functional role	Molecular mechanism	Related genes	Refs.
Digestive system	Colorectal cancer	Writer	DKC1	Upregulation	Oncogene	Poor	Binds and enhances ribosomal protein expression to promote cancer progression	DKC1 binds ribosomal proteins that interact with HRAS to inhibit the downstream RAS/RAF/MEK/ERK pathway.	HRAS	496
		Writer	DKC1	Upregulation	/	/	/	/	/	491
		Writer	DKC1	Upregulation	Oncogene	Poor	Associated with TNM stage and lymph node metastasis	Direct activation of HIF-1α	HIF-1α	498
	Hepatocellular carcinoma	Writer	DKC1	Upregulation	Oncogene	Poor	Associated with hepatitis B surface antigen status, serum AFP, and advanced clinical stage	Associated with MYC and MKI67 expression, may be involved in tumorigenic processes	MYC, MKI67	499
		Writer	DKC1	Upregulation	Oncogene	Poor	Promotes tumor cell survival	PDIA3 increases DKC1 mRNA levels	PDIA3	500
	Gastric cancer	Writer	DKC1	Upregulation	/	/	/	/	/	501
Non-digestive system cancer	Breast cancer	Writer	DKC1	/	Oncogene	/	Low expression of DKC1 reduces telomerase activity and rRNA pseudo uridylation	/	/	506
		Writer	DKC1	Upregulation	Oncogene	Poor	Associated with tumor grade, nucleolar score, and Nottingham Prognostic Index	/	/	505
	Non-small cell lung cancer	Writer	DKC1	/	Oncogene	/	Promotes proliferation and migration, inhibits apoptosis	Synergizes with PCAT1 via the VEGF/AKT/Bcl2/Caspase9 pathway	LncRNA PCAT1	510
		Writer	DKC1	/	/	Poor (only for TERC-negative tumors)	/	/	/	512
		Writer	PUS10	Upregulation	/	/	Promotes immortalization of tumor cells	rs9309336 may interfere with PUS10 expression and reduce tumor cell sensitivity to TRAIL	rs9309336	513
	Prostate cancer	Writer	DKC1	Upregulation	Oncogene	/	Moderately associated with hTR and MKI67, Promotes proliferation	/	/	511
	Glioblastoma	Writer	PUS7	Upregulation	Oncogene	Poor	Promotes cell growth and self-renewal	Inhibits tRNA pseudo uridylation	/	515
		Writer	DKC1	Upregulation	Oncogene	Poor	Promotes proliferation, invasion, migration	/	/	516
	Oral squamous cell carcinomas	Writer	DKC1	Upregulation	/	/	Associated with active cell proliferation	/	/	517
	Ovarian Cancer	Writer	PUS7	Upregulation	Oncogene	/	Promotes proliferation	/	/	518

### Ψ and digestive system cancers

As early as 1983, Salvatore et al.^493^ pointed out that Ψ in serum can be used as a tumor marker. In 1988, studies reported that serum Ψ and α-fetoprotein (AFP) could serve as complementary markers for HCC diagnosis. Among seven patients with very small liver cancer, four were negative for AFP but positive for serum Ψ, suggesting that serum Ψ may be a useful marker for early diagnosis of HCC.^494^ In addition, detection of Ψ in urine also is used as a marker for some tumors, including CRC and HCC.

Turano et al.^495^ detected DKC1 mRNA expression in cancer and adjacent tissues of eight CRC patients using real-time polymerase chain reaction. Results supported that DKC1 expression can be used as a tumor marker for CRC. In colon cancer, DKC1 is highly expressed and predicts a poor prognosis. DKC1 binds to and increases the expression of some ribosomal proteins in a manner dependent on its Ψ synthase activity. The latter interacts with HRAS to inhibit the downstream RAS/RAF/MEK/ERK pathway. The DKC1 inhibitor pyrazofuran and the MEK1/2 inhibitor trametinib synergistically inhibit CRC growth.^496^ Studies also found a cancer-specific single nucleotide variant at nucleotide 1248.U in 18S rRNA from CRC patients. Loss of rRNA m^1^acp^3^Ψ modification is a hallmark of cancer.^497^ Furthermore, DKC1 enhances CRC angiogenesis by directly activating hypoxia-inducible factor-1α transcription, promoting CRC cell metastasis.^498^ In HCC, DKC1 expression is similarly upregulated and exerts a tumor-promoting effect. High DKC1 expression was an independent prognostic factor (hazard risk = 2.912; *P* = 0.007). Also, DKC1 expression was significantly correlated with MKI67 and MYC mRNA.^499^ This may involve the molecular mechanism of DKC1 promoting cancer. Of course, further research is needed in the future. Furthermore, oxidatively modified protein disulfide isomerase-related 3 increases DKC1 mRNA levels and tumor cell survival, driving the progression of liver malignancies.^500^ H/ACA snoRNA SNORA24 mediates the pseudouridylation of rRNA U609 and U863. Translation efficiency and accuracy are reduced in HCC cells lacking SNORA24-directed Ψ modification.^501^

### Ψ and nondigestive system cancers

Ψ in nondigestive cancers is currently focused on breast, lung, and prostate cancers. Studies successfully revealed and summarized the predictive value of Ψ in these cancers, and Ψ is expected to become a novel cancer biomarker.^435,502–504^ Nuclear and nucleolar expression of DKC1 protein was strongly associated with higher tumor grade, high nucleolar score, and poorer Nottingham prognostic index. Furthermore, DKC1 overexpression in BRC predicts a poor prognosis.^505^ Dyskerin expression is highly variable in sporadic human tumors of various histological origins. Particularly in BRC, low dyskerin expression results in rRNA pseudouridylation and a reduction in the RNA component of telomerase.^506^ In addition, p53 is a well-known tumor suppressor, and p53 function is also reduced in the presence of low keratin levels, contributing to the tumor phenotype.^507^ At first glance, the finding that DKC1 is overexpressed in common carcinomas seems contradictory, as keratin-inactivating mutations in DC confer increased tumor susceptibility. In tumors arising from DC, a possible explanation is that keratin mutations would constitute the major event and favorably trigger subsequent cancer development. Conversely, dyskerin overexpression may represent an increase in RNA biosynthesis and telomerase activity in cancer progression, such as in breast cancer.^508,509^ In NSCLC, lncRNAs PCAT1 and DKC1 act synergistically to promote cancer cell proliferation and invasion and inhibit cancer cell apoptosis. The molecular mechanism involves the vascular endothelial growth factor/Akt/Bcl-2/caspase-9 pathway.^510^ Similarly, in prostate cancer, DKC1 is also highly expressed and promotes cancer progression. Unlike NSCLC, DKC1 mainly affects prostate cancer cell proliferation without causing apoptosis, highlighting the important role of DKC1 in maintaining protein biosynthesis.^511^ In another interesting study, an association between dyskerin expression and survival was found in lung cancer only in the absence of amplification of the *TERC* gene. Overall survival was significantly reduced in patients with higher dyskerin expression. In conclusion, the effect of dyskerin expression on tumor clinical outcome is related to its role in maintaining TERC stability.^512^ In addition, one study investigated the relationship between single nucleotide polymorphisms in chromatin-interacting regions and lung cancer risk and found four new lung cancer susceptibility loci. rs9309336 may interfere with PUS10 expression, reducing tumor cell sensitivity to tumor necrosis factor-related apoptosis-inducing ligand (TRAIL). Finally, it promotes tumor cell immortalization and lung cancer occurrence.^513^ Jana et al.^514^ previously showed that PUS10 moved to the mitochondria during TRAIL-induced apoptosis, releasing cytochrome c and SMAC. This CRM1-mediated nuclear export of PUS10 needed caspase-3, and the translocated PUS10 reciprocally activated caspase-3 to form an amplification loop. Anything that interfered with HuP10 movement or its interaction with mitochondria reduced tumor cell sensitivity to TRAIL. The effect of the PUS enzyme family in cancer is still less studied, and more research is worth adding in the future.

Other studies on Ψ in glioma,^515,516^ OSCC,^517^ and OC^518^ are not described in detail in the main text and can be found in Table 3.

## Impact of RNA methylation modification on cancer therapy

DNA methylation was discovered initially. With the advancement of NGS technology, RNA methylation appeared in people’s field of vision. Over the years, there has been a relatively good understanding of some of the basics of RNA methylation. It mainly includes the common types of RNA methylation modifications, such as m^6^A, m^5^C, and Ψ, and the types and functions of the regulators of RNA methylation modification. More attention is paid to the impact of RNA methylation modification on life activities and disease progression in recent years, especially in cancer. However, despite extensive research, the impact on the mechanisms by which RNA modifications and their associated proteins are regulated in cancer remains largely unknown. Growing evidence suggested that RNA modification pathways are also mis regulated in human cancers, which may be ideal targets for cancer therapy. Numerous factors supported the idea that RNA-modifying regulators may be ideal targets for drug discovery, and developing activators or inhibitors of RNA methylation-modifying regulators could add valuable drugs and experience for cancer treatment.

In the following, this review mainly discussed and summarized the role of m^6^A in cancer therapy. A summary of the impact of m^5^C and Ψ modifications in cancer therapy is presented, although relevant studies are still relatively few. m^5^C methyltransferases NSUN3 and DNMT2 can mediate the generation of 5-AZA-sensitive chromatin structures through a series of mechanisms, providing new insights into drug resistance in leukemia treatment.^429^ In addition to the effects on chemoresistance, therapeutic approaches targeting m^5^C modulators may also hold great promise. Notably, m5C is the most common form of DNA methylation. However, there is no way to control the specific inhibition of RNA methylase by drugs without affecting DNA methylation. As mentioned earlier, given that Ψ is present in urine, blood, saliva, etc., it is expected to be a potential biomarker for early cancer diagnosis.^519,520^ Moreover, this noninvasive detection method has huge clinical application prospects. In addition, pyrazoline and 5-fluorouracil are currently two common drugs that inhibit DKC1, opening the understanding of the role of Ψ in cancer therapy. Of course, further in-depth research and the development of new targeted drugs are needed.

m^6^A is the most common type of RNA methylation modification. m^6^A modification is regulated by methyltransferases, demethylases, and RNA-binding proteins. m^6^A modulators can be used as potential therapeutic targets for cancer and in targeted therapy, radiotherapy and chemotherapy, immunotherapy, and other aspects. In terms of targeted therapy, there is no small-molecule inhibitor of RNA methyltransferase, but some inhibitors of RNA demethylase have been developed and used.^304,308,521–528^ Current inhibitors primarily target FTO, a common demethylase. For example, MO-I-500 can selectively inhibit the m^6^A demethylase activity of FTO. MO-I-500 is an α-ketoglutarate mimetic that exhibits high specificity for FTO. It inhibits FTO in vitro and in vivo, as manifested by increased overall levels of RNA methylation. After MO-I-500 inhibited FTO, it inhibited the survival of rare pan-drug-resistant triple-negative inflammatory breast cancer cells.^521,522^ Meclofenamic acid (MA) is a nonsteroidal anti-inflammatory drug approved by the U.S. Food and Drug Administration. Recent studies revealed that MA could specifically inhibit FTO, increasing m^6^A levels. In GBM, FTO inhibition using MA2, the ethyl ester form of MA, inhibits cancer cell survival and tumor progression.^304,523^ R-2HG is the major metabolite of mutant isocitrate dehydrogenase 1/2. Also considered an FTO inhibitor, R-2HG exerts a tumor suppressor effect in leukemia and glioma by targeting FTO/m^6^A/MYC/CEBPA signaling.^308^ In addition, FB23-2, rhein, etc., are currently known inhibitors of FTO.^524,525^ In chemoradiotherapy, resistance to therapy is an unresolved bottleneck and challenge in cancer treatment. Several studies showed that RNA modifications affect primary and acquired drug resistance in cancer. m^6^A modulators also reduce or exacerbate resistance to chemoradiotherapy in cancer patients.^168^ For example, targeting SNHG3/miR-186-5p reversed platinum treatment-induced elevated m^6^A levels by modulating METTL3 in esophageal cancer.^529^ m^6^A modification of FZD10 mRNA leads to PARPi resistance in BRCA-deficient epithelial ovarian cancer cells by upregulating the Wnt/β-catenin pathway.^530^ In addition, METTL3 can promote chemoresistance and radioresistance of pancreatic cancer cells.^531^ In terms of immunotherapy, two studies also revealed that m^6^A might be related to cancer immunotherapy. Yang et al.^327^ found that FTO promotes anti-PD-1 resistance, and inhibition of FTO expression can increase melanoma cell sensitivity to immunotherapy. Li et al.^532^ found that m^6^A-modifying enzymes can act as key regulators of T cells, regulating T-cell homeostasis. In all, the impact of RNA methylation modification in cancer treatment is very extensive, and there are still many unexplored areas with huge research prospects.

## Conclusion and prospects

To date, at least 170 different posttranscriptional RNA modifications are known.^533,534^ These modifications range from methylation to complex chemical structures, with methylation being the most abundant. With the development of high-throughput NGS technology, the improvement of the sensitivity of liquid chromatography, and the update of other sequencing technologies, there is a better understanding and mastery of identifying the overall level of RNA methylation. The discovery and functional studies on the types of RNA methylation and various methylation-related regulators have greatly advanced the understanding of RNA methylation. RNA methylation plays an indispensable role in regulating gene transcription, expression, editing, stability, and degradation.^535^ RNA modification is a key player in various cellular biological processes, in which RNA modification enzymes play a decisive role.^464^ As mentioned above, epigenetic modification refers to heritable phenotypic changes without changing the nucleic acid sequence. These changes include DNA methylation, histone modifications, chromatin remodeling, and RNAi. DNA methylation and histone modifications mainly affect transcriptional events, whereas reversible RNA methylation mainly affects the regulation of posttranscriptional gene expression and directly affects protein production.^144^ RNA modifications can affect not only normal biological processes, including development and cell differentiation, but also abnormal life activities, such as inflammation, infertility, neurological diseases, and cancer. RNA modifications and their regulators are often aberrantly expressed in tumor tissues. Abnormal expression is also closely related to the prognosis of cancer patients. The effects of RNA modifications in cancer are multiple. Both can play a role in promoting cancer. On the one hand, RNA modification can reduce the stability of tumor suppressor genes to eliminate their inhibitory effect, thereby promoting cancer progression; on the other hand, RNA modification can enhance the stability and stability of proto-oncogene transcripts. Conversely, they can also play a tumor suppressor role in cancer, inhibiting cancer cell proliferation, migration, and invasion and inhibiting cancer occurrence and development. In some cases, an enzyme may have opposing effects in different cancer types or even very different effects within the same cancer type. Because RNA modifications are diverse, and the number of modified coding RNAs and ncRNAs is enormous, this paradox seems not surprising. The current hypothesis is based only on individual cohorts with a remarkably limited number of cancer patients whose genetic background and environmental factors influence the results. Based on this, this review affirmed the importance of developing small-molecule inhibitors targeting RNA modification sites and RNA-modifying enzymes, providing new and more targeted approaches for cancer therapy. In addition, more relevant studies are needed to further verify and explain the specific mechanism of RNA methylation in cancer and explain some of the existing contradictory studies more reasonably.
